# Mitapivat reprograms the RBC metabolome and improves anemia in a mouse model of hereditary spherocytosis

**DOI:** 10.1172/jci.insight.172656

**Published:** 2023-10-23

**Authors:** Alessandro Matte, Anand B. Wilson, Federica Gevi, Enrica Federti, Antonio Recchiuti, Giulia Ferri, Anna Maria Brunati, Mario Angelo Pagano, Roberta Russo, Christophe Leboeuf, Anne Janin, Anna Maria Timperio, Achille Iolascon, Elisa Gremese, Lenny Dang, Narla Mohandas, Carlo Brugnara, Lucia De Franceschi

**Affiliations:** 1 Department of Medicine, University of Verona, and Azienda Ospedaliera Universitaria Verona, Policlinico GB Rossi, Verona, Italy.; 2 Department of Ecological and Biological Sciences, University of Tuscia, Viterbo, Italy.; 3 Department of Medical, Oral, and Biotechnology Science, “G.d’Annunzio” University of Chieti – Pescara, Center for Advanced Studies and Technology, Chieti, Italy.; 4 Department of Molecular Medicine, University of Padua, Padua, Italy.; 5 Dipartimento di Medicina Molecolare e Biotecnologie Mediche, Università degli Studi di Napoli Federico II, Naples, Italy.; 6 CEINGE Biotecnologie Avanzate, Naples, Italy.; 7 INSERM, Paris, France.; 8 Université Paris 7 — Denis Diderot, Paris, France.; 9 Assistance Publique — Hôpitaux de Paris, Hôpital Saint-Louis, Paris, France.; 10 Division of Clinical Immunology, Fondazione Policlinico Universitario A. Gemelli–Istituto di Ricovero e Cura a Carattere Scientifico (IRCCS), Università Cattolica del Sacro Cuore, Rome, Italy.; 11 Immunology Core Facility, Fondazione Policlinico Universitario A. Gemelli–IRCCS, Rome, Italy.; 12 Agios Pharmaceuticals Inc., Cambridge, Massachusetts, USA.; 13 New York Blood Bank Center, New York, New York, USA.; 14 Department of Laboratory Medicine, Boston Children’s Hospital, Boston, Massachusetts, USA.; 15 Department of Pathology, Harvard Medical School, Boston, Massachusetts, USA.

**Keywords:** Hematology, Therapeutics, Genetic diseases, Mouse models

## Abstract

Hereditary spherocytosis (HS) is the most common, nonimmune, hereditary, chronic hemolytic anemia after hemoglobinopathies. The genetic defects in membrane function causing HS lead to perturbation of the RBC metabolome, with altered glycolysis. In mice genetically lacking protein 4.2 (4.2^–/–^; Epb42), a murine model of HS, we showed increased expression of pyruvate kinase (PK) isoforms in whole and fractioned RBCs in conjunction with abnormalities in the glycolytic pathway and in the glutathione (GSH) system. Mitapivat, a PK activator, metabolically reprogrammed 4.2^–/–^ mouse RBCs with amelioration of glycolysis and the GSH cycle. This resulted in improved osmotic fragility, reduced phosphatidylserine positivity, amelioration of RBC cation content, reduction of Na/K/Cl cotransport and Na/H-exchange overactivation, and decrease in erythroid vesicles release in vitro. Mitapivat treatment significantly decreased erythrophagocytosis and beneficially affected iron homeostasis. In mild-to-moderate HS, the beneficial effect of splenectomy is still controversial. Here, we showed that splenectomy improves anemia in 4.2^–/–^ mice and that mitapivat is noninferior to splenectomy. An additional benefit of mitapivat treatment was lower expression of markers of inflammatory vasculopathy in 4.2^–/–^ mice with or without splenectomy, indicating a multisystemic action of mitapivat. These findings support the notion that mitapivat treatment should be considered for symptomatic HS.

## Graphical abstract

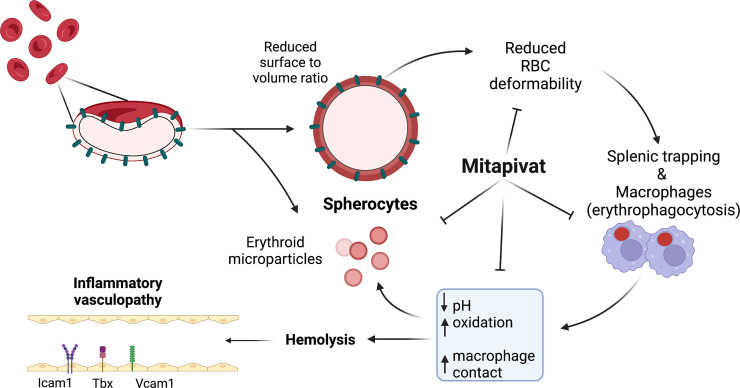

## Introduction

Hereditary spherocytosis (HS) is the most common, inherited, nonimmune chronic hemolytic anemia after hemoglobinopathies. It is characterized by a wide heterogeneity in clinical expression with causative genetic variants in 5 genes (*ANK1*, *SPTA1*, *STPB*, *SLC4A1*, and *EPB42*). It is inherited either dominantly or recessively, with a notable association with de novo mutations (1–3). All the HS-related genes encode membrane proteins that are key components of the membrane skeleton and play a role in regulating membrane function. Clinically, a broad phenotypic spectrum is recognized, ranging from asymptomatic or well-compensated anemia to severe forms requiring regular blood transfusions and splenectomy (4).

The pathogenetic mechanism of HS relies on loss of membrane surface and resultant decreased membrane surface area to volume ratio leading to increased cell sphericity and decreased cellular deformability. The sequestration of the nondeformable spherocytes in the spleen and their subsequent phagocytosis by the splenic macrophages is responsible for anemia and splenomegaly. Once trapped in the spleen, the abnormal erythrocytes undergo splenic conditioning, resulting in further loss of surface area. Low pH, low concentrations of glucose and adenosine triphosphate, contact of erythrocytes with macrophages, and high local concentrations of oxidants contribute to splenic conditioning. Some of these conditioned erythrocytes escape the hostile environment of the spleen, re-enter the systemic circulation, and account for the tail of fragile cells in osmotic fragility tests (2, 5, 6).

Metabolic profiling of dried blood spots from patients with HS identified significant features that suggest a glycolytic disturbance, including a decrease in glyceraldehyde-3-phosphate, a glycolytic intermediate, and 2,3-bisphosphoglycerate, which is produced via conversion of 1,3-bisphosphoglycerate, another metabolite of glycolysis, in the Luebering–Rapoport glycolytic shunt and is responsible for enhancing oxygen dissociation from hemoglobin (Hb) (4). The final step of the glycolytic pathway depends on pyruvate kinase (PK), whose activity is decreased in HS and is accompanied by a loss of membrane-bound PK (7). These metabolic changes contribute to worsening RBC membrane cohesion by functionally thwarting the membrane lipid bilayer and cytoskeletal protein interactions, defects that underlie HS and other hemolytic anemias (4, 7–13).

In humans, 4 different PK isoforms have been identified: PKr in RBCs; PKm1 in brain and heart; PKl in kidney and liver; and PKm2 in intestine cells (14). The orally active PK activator mitapivat is the first approved therapeutic agent that is clinically beneficial for effective treatment of patients with PK deficiency (15, 16).

Recent evidence in a murine mouse model for β-thalassemia has shown that mitapivat treatment decreased anemia and reduced ineffective erythropoiesis in conjunction with amelioration of iron homeostasis (17). These preclinical studies in a β-thalassemic mouse model generated the rationale for a phase II proof-of-concept study (ClinicalTrials.gov NCT03692052) of mitapivat in patients with nontransfusion-dependent thalassemias (NTDTs), including both α- and β-thalassemia genotypes. Kuo et al. showed that mitapivat improved anemia and reduced markers of ineffective erythropoiesis and hemolysis in patients with NTDTs (18, 19). More recently another PK activator (FT-4202; etavopivat) has been developed (20, 21). Mitapivat and etavopivat have also been tested in patients with sickle cell disease (SCD), another RBC disorder characterized by a relative PK deficiency. Both PK activators have been reported to ameliorate hemolysis in mouse models of SCD and in proof-of concept studies in small cohorts of patients with SCD (22, 23). These findings suggest that PK activators could be potential novel therapeutic options for management of other hereditary RBC disorders such as HS characterized by excess oxidative stress, perturbation of the glycolytic pathway, and/or relative PK deficiency. In this regard, PK activity enhancement can be thought to mitigate the instability of the HS RBC membrane by replenishing ATP stores that are then used in the ion-pump activities (24), acceleration of glutathione (GSH) synthesis, and a feed-forward loop for glycolysis itself (7, 25, 26). Thus, in HS, PK activity seems to be inadequate to support energy demand of circulating erythrocytes. Indeed, previous studies have shown an inverse correlation between PK activity and reticulocyte counts or the reduction in eosin-5′-maleimide binding or the mount of spherocytes in patients with HS (27–29)

In the present study, we tested the effectiveness of mitapivat in a mouse model of HS and show that mitapivat metabolically reprograms RBCs in HS. Importantly, we found the noninferiority of mitapivat versus splenectomy in decreasing anemia in this setting. Our results support the consideration of mitapivat as a potential new therapeutic agent for HS.

## Results

### Treatment with mitapivat reprograms the metabolic profile of RBCs and improves anemia in a mouse model for HS.

**Figure 1:**
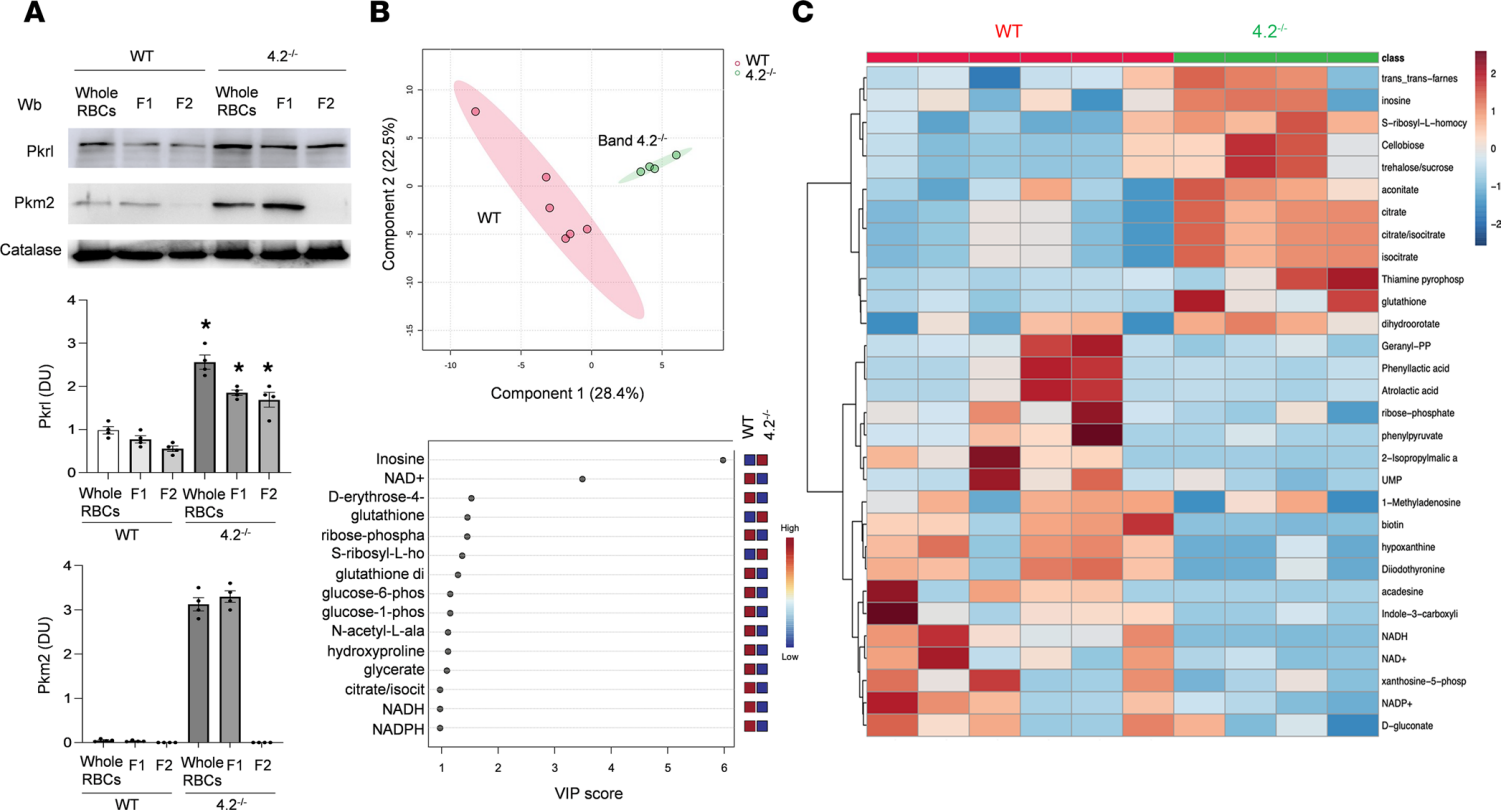
RBCs from 4.2^–/–^ mice are characterized by an abnormal metabolomic profile. (**A**) IB analysis using specific Abs against Pklr and Pkm2 in unfractionated RBCs (whole RBCs) and fractionated RBCs, according to density in F1(density N1.074, corresponding to a young and reticulocyte-enriched fraction) and F2 (density N1.092, corresponding to older RBCs) from WT and 4.2^–/–^ mice. Protein (75 μg) was loaded on an 8% T, 2.5%C polyacrylamide gel; catalase was the protein loading control. One representative gel from 3 with similar results is shown. Densitometric analysis of IBs is shown in the bar graphs. Data are reported as mean ± SEM (*n* = 4). *P < 0.05 compared with WT animals by 1-way ANOVA. (**B**) 2D PCA scores plot demonstrating statistical clustering of WT and 4.2^–/–^ RBC metabolomic profiles (*n* = 4–6). The 15 metabolites contributing most to the separation of groups are reflected by high variable importance in projection (VIP) scores (bottom graph). These metabolites include intermediates of glycolysis, TCA, and GSH pathways. (**C**) Heatmap of the 30 most significant different features identified by *t* test (*P* < 0.005; *n* = 4–6). The heatmap scale ranges from –2 to 2 (Kyoto Encyclopedia of Genes and Genomes pathway metabolites) was expressed on a log2 scale. Figures were created using MetaboAnalyst 5.0. Wb: Western-blot; DU: density unit; VIP: variable importance in projection; S-ribosyl-L- ho, S-ribosyl-L- homocysteine; Geranyl pyrophosphate a, Geranyl-pyrophosphate 2-isopropylmatic acid.

The 4.2^–/–^ mice exhibit a hematological phenotype comparable to that of mild HS, which represents the phenotype of the majority of patients with this membrane disorder (1, 3). Mouse models of HS due to either ankyrin, spectrin, or band 3 deficiency show a more severe hematologic phenotype, similar to that of individuals with transfusion-dependent HS (30, 31). We first evaluated the expression of Pkr and Pkm2 isoforms in RBCs from 4.2^–/–^ mice. We analyzed the entire population of RBCs as well as 2 subpopulations of density-fractionated RBCs. Fraction 1 (F1) was enriched in reticulocytes and fraction 2 (F2) was enriched for the densest RBCs, representing aged erythrocytes (17). As shown in Figure 1A, the expression of Pkr was higher in both F1 and F2 RBC fractions from 4.2^–/–^ mice compared with WT RBCs**.** In contrast, the expression of Pkm2 was increased only in F1 from 4.2^–/–^ mice (Figure 1A). Consistent with expression levels, PK activity was elevated in 4.2^–/–^ RBCs compared with WT erythrocytes (Supplemental Figure 1; supplemental material available online with this article; https://doi.org/10.1172/jci.insight.172656DS1).

Metabolomic analysis of RBCs from 4.2^–/–^ and WT mice was performed using a liquid chromatography–mass spectrometry approach (32, 33) (Figure 1, B and C). The principal component analysis (PCA) effectively and distinctly separated the 2 groups (*x* axis in Figure 1B), suggesting that the absence of protein 4.2 caused a significant change in the overall metabolite profile compared with healthy erythrocytes (Figure 1B). The major metabolite contributors to the separation of the 2 groups in PCA are identified by high variable importance in projection scores (Figure 1B). The metabolites identified as significantly different by the *t* test analysis included (a) glucose-6-phosphate and 2,3-bisphosphoglycerate, the first product of glycolysis and the product of the Rapoport–Luebering shunt, respectively; (b) the molecules involved in the GSH pathway, such as GSH and NADPH; and (c) citrate. The 15 features that contributed most to the separation of WT and 4.2^–/–^ mouse RBCs are displayed in Figure 1C and Supplemental Figure 2.

**Figure 2:**
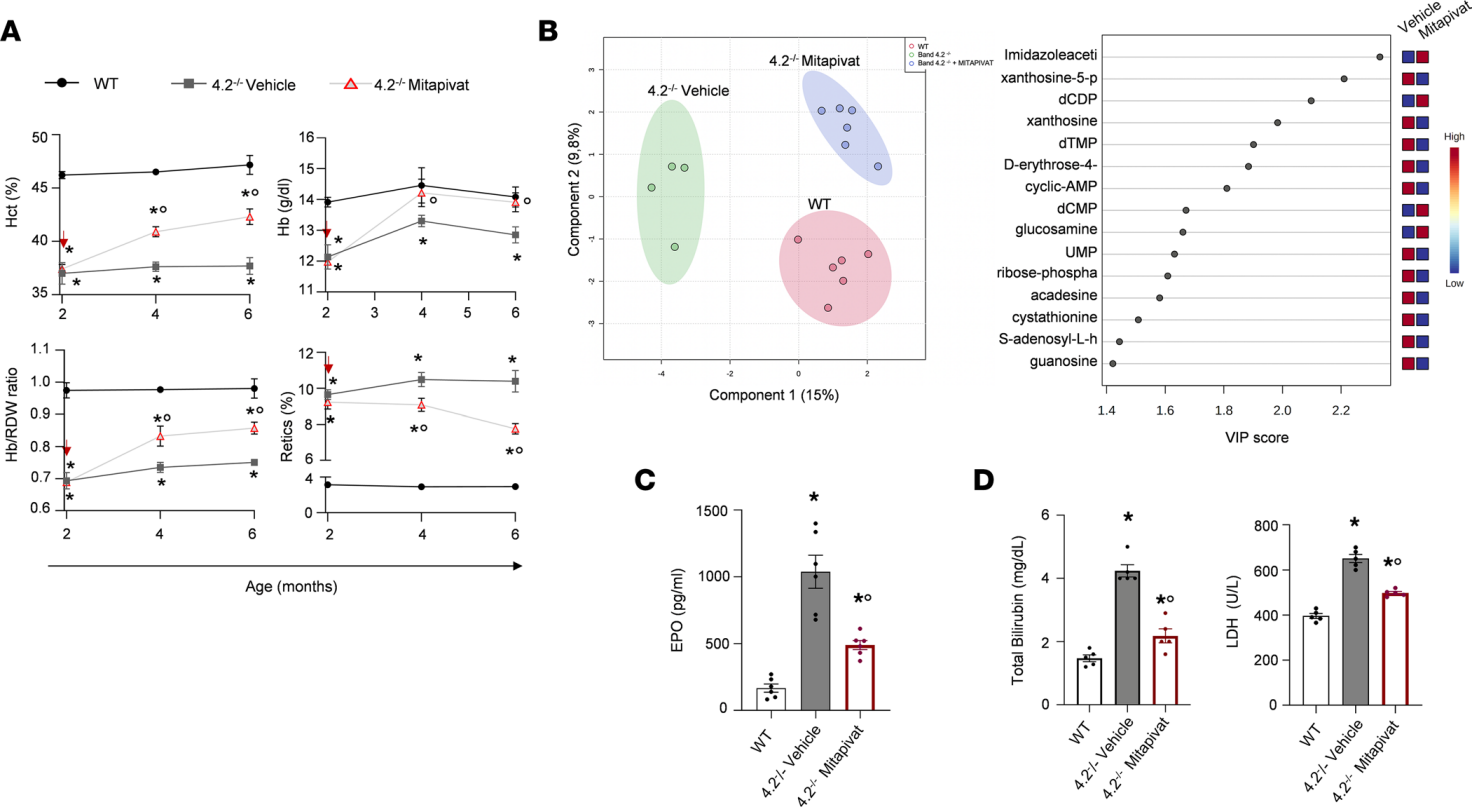
Mitapivat improves anemia by metabolic reprogramming of 4.2^–/–^ mouse RBCs. (**A**) Hct, Hb, and Hb to RDW ratio, as marker of spherocytosis and reticulocyte count in WT and 4.2^–/–^ mice treated with vehicle or mitapivat (100 mg/kg/d) up to 6 months of age. The red arrow indicates the starting point for mitapivat administration. Data are reported as mean ± SEM (WT, *n* = 5; 4.2^–/–^ vehicle, *n* = 9; 4.2^–/–^ mitapivat, *n* = 12). *P < 0.05 compared with WT; °*P* < 0.05 compared with vehicle-treated 4.2^–/–^ mice by 1 way ANOVA with Dunnett’s longitudinal comparison. (**B**) 2D PCA scores obtained from the analysis of untargeted metabolites of RBCs from WT and 4.2^–/–^ mice treated with vehicle or mitapivat (100 mg/kg/d) for 6 months. The value of each biological replicate was normalized. Data on WT animals are in red, on 4.2^–/–^ mice are in green, and on mitapivat-treated 4.2^–/–^ mice are in blue (*n* = 4–6). The variable importance in projection (VIP) score plot for the top 15 most important metabolite features identified by partial least squares discriminant analysis (PLS-DA). The box indicates the relative concentration from vehicle and mitapivat groups (right panel). Figures were created using MetaboAnalyst 5.0. (**C** and **D**) Plasma EPO, total bilirubin, and LDH, as markers of hemolysis, in WT and 4.2^–/–^ mice treated with vehicle or mitapivat (100 mg/kg/d) for 6 months (*n* = 5). *P < 0.05 compared with WT; °*P* < 0.05 compared with vehicle-treated 4.2^–/–^ mice by 1-way ANOVA. dCMP, deoxycytidine monophosphate; dTMP, deoxytimidine monophosphate; AMP, adenosine monophosphate; UMP, uridine 5-monophosphate.

Mitapivat was added to the 4.2^–/–^ mouse diet for up to 6 months to minimize the possible stress related to animal manipulation, as previously reported by Matte et al. (17). Treatment of 4.2^–/–^ mice with mitapivat significantly increased hematocrit (Hct) and Hb values, and Hb to RBC distribution width (RDW) ratio, a complementary RBC index for spherocytosis and dense RBCs, when compared with vehicle-treated animals (Figure 2A). The increase in Hct in 4.2^–/–^ mice was associated with lower reticulocyte counts (Figure 2A). Similar results were observed in 4.2^–/–^ mice treated with mitapivat for either 5 or 6 months (Supplemental Figure 3, A and B). Metabolomic analysis highlighted a shift of the RBC profile toward that of WT erythrocytes (Figure 2B). We found increased 2-phosphoglycerate and decreased GSH and NADP^+^ levels, suggesting that mitapivat metabolically reprograms 4.2^–/–^ mouse RBCs. This was associated with a significant increase in RBC ATP content (Supplemental Figure 3C). Indeed, mitapivat, by PK activation, overcomes the abnormal compartmentalization of ATP into cytosolic and membrane-bound pools observed in HS (7). Mitapivat re-establishes a more physiologic metabolic profile of 4.2^–/–^ RBCs, characterized by the balance between glycolysis and the pentose phosphate pathway combined with the GSH system (7, 34).

Anemia improvement in 4.2^–/–^ mice treated with mitapivat was associated with a significant reduction in spleen to BW ratio (Supplemental Figure 4, A and B) and decreased spleen iron content (Supplemental Figure 4B). Treatment of 4.2^–/–^ mice with mitapivat was also associated with a trend toward a decrease in splenic erythropoietic activity and a significant reduction in BM erythropoiesis (Supplemental Figure 4, C and D). This was mainly related to the reduction in populations III and IV, corresponding, respectively, to polychromatic erythroblasts and orthochromatic erythroblasts (Supplemental Figure 4E). A similar trend was also observed in splenic erythroblast populations III and IV (Supplemental Figure 4E).

Lower plasma erythropoietin (EPO) levels in 4.2^–/–^ mice treated with mitapivat and significant decreases in total bilirubin and lactate dehydrogenase (LDH) concentrations support the notion that mitapivat treatment decreases chronic hemolysis (Figure 2D).

Collectively, our data indicate that the improvement of the hematologic phenotype observed in 4.2^–/–^ mice treated with mitapivat is accompanied by reprogrammed metabolic profile.

### Mitapivat ameliorates 4. 2^–/–^ mouse RBC features and reduces erythroid vesicles release.

**Figure 3:**
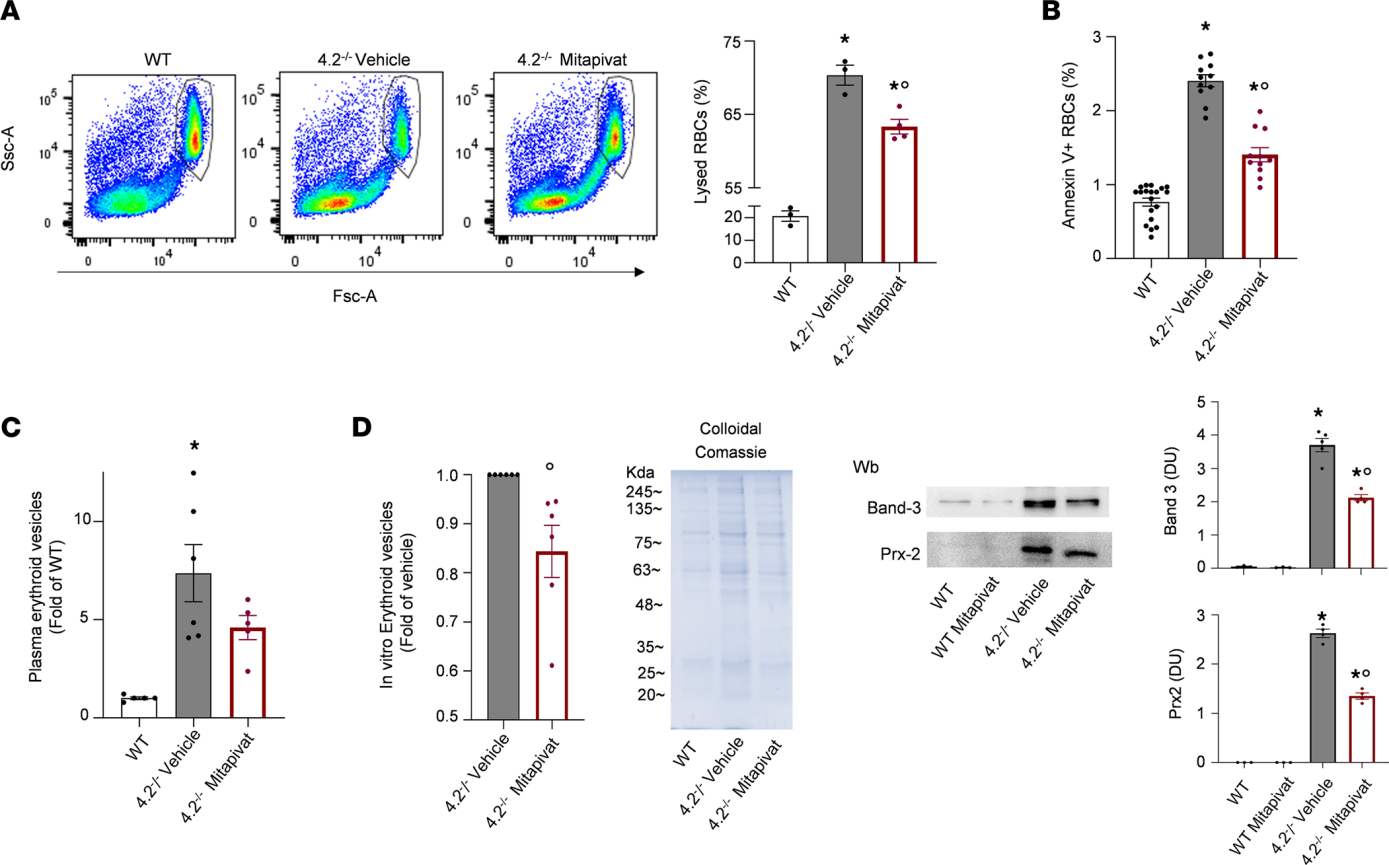
Mitapivat improves RBC osmotic fragility, reduces the amount of circulating annexin-V^+^ erythrocytes, and decreases the release of erythroid vesicles in 4.2^–/–^ mice. (**A**) Representative scatter plots (left panel) of the osmotic fragility test determined by flow cytometry and percentage of RBC lysis (right panel) at 192 mOsm of RBCs from WT and 4.2^–/–^ mice treated with either vehicle or mitapivat (100 mg/kg/d) for 6 months. Results are reported as mean ± SEM from 3–4 mice/group. *P < 0.05 compared with WT; °*P* < 0.05 compared with vehicle-treated 4.2^–/–^ mice by 1-way ANOVA. Fsc, forward scatter; Ssc, side scatter. (**B**) Annexin-V^+^ RBCs from WT and 4.2^–/–^ mice treated with either vehicle or mitapivat (100 mg/kg/d) for 6 months. Results are mean ± SEM from 11–19 mice/group. *P < 0.05 compared with WT; °*P* < 0.05 compared with vehicle-treated 4.2^–/–^ mice by 1-way ANOVA. (**C**) Plasma erythroid microvesicles determined by flow cytometry from WT and 4.2^–/–^ mice treated with either vehicle or mitapivat (100 mg/kg/d) for 6 months. Results are reported as mean ± SEM from 5–6 mice/group. *P < 0.05 compared with WT by *t* test. (**D**) The bar chart shows flow cytometric analysis results of erythroid vesicles in vitro released under shared stress conditions from 4.2^–/–^ RBCs incubated for 50 minutes in the presence of vehicle or mitapivat (2 μM). Results are reported as mean ± SEM; *n* = 6. °*P* < 0.05 compared with vehicle-treated mice, determined by *t* test. Representative Coomassie-stained gel and IB analysis using specific Abs against band 3 and peroxiredoxin-2 (Prx-2) of erythroid vesicles in vitro released under shared stress conditions from WT and 4.2^–/–^ erythrocytes incubated for 50 minutes in the presence of either vehicle or mitapivat (2 μM). Results are reported mean ± SEM; *n* = 3–5. °*P* < 0.05 compared with vehicle-treated RBCs, determined by *t* test. Wb, Western blot; DU: density unit.

The decreased cell surface area to volume ratio and increased cell sphericity with attendant increased osmotic fragility observed in HS erythrocytes are accompanied by externalization of phosphatidylserine (1, 35). Mitapivat treatment of 4.2^–/–^ mice ameliorates RBCs’ osmotic fragility (Figure 3A) and decreases the amount of annexin-V^+^ cells when compared with 4.2^–/–^ controls (Figure 3B). These changes were associated with a trend in decreased numbers of circulating erythroid vesicles in mitapivat-treated 4.2^–/–^ mice compared with 4.2^–/–^ controls (Figure 3C). Because the numbers of erythroid vesicles measured ex vivo can be affected by hypersplenism (32, 33), we evaluated the in vitro generation of erythroid vesicles from RBCs of 4.2^–/–^ mice in vivo treated with or without mitapivat.

As shown in Figure 3D, mitapivat significantly reduced the in vitro release of erythroid vesicle from 4.2^–/–^ mouse erythrocytes and normalized the protein composition of released erythroid vesicles (Figure 3D). Mitapivat significantly reduced the amount of band 3 and membrane associated peroxiredoxin-2 (Figure 3D) in the released vesicles, which constitute the protein machinery involved in generation of erythroid vesicles (36, 37).

The general improvement of RBC features was also supported by the amelioration of RBC cation content (Supplemental Figure 5A). Because we previously showed that the overactivation of both Na/K/Cl cotransport and Na/H exchange were mainly responsible for abnormalities in 4.2^–/–^ mouse RBCs cation content (31), we evaluated both cation transport systems in RBCs from 4.2^–/–^ mice treated with either vehicle or mitapivat. As shown in Supplemental Figure 5, B and C, we observed lower Na/K/Cl cotransport and Na/H exchange activities in RBCs from mitapivat-treated 4.2^–/–^ mice when compared with vehicle-treated animals, suggesting an improved RBC ion homeostasis related to the amelioration of RBC membrane mechanical stability.

Taken together our data indicate that mitapivat significantly ameliorates the membrane mechanical cohesion and stability of 4.2^–/–^ mouse RBCs.

### Mitapivat-treated 4. 2^–/–^ mice have reduced splenic conditioning and improved vascular inflammatory biomarkers.

**Figure 4:**
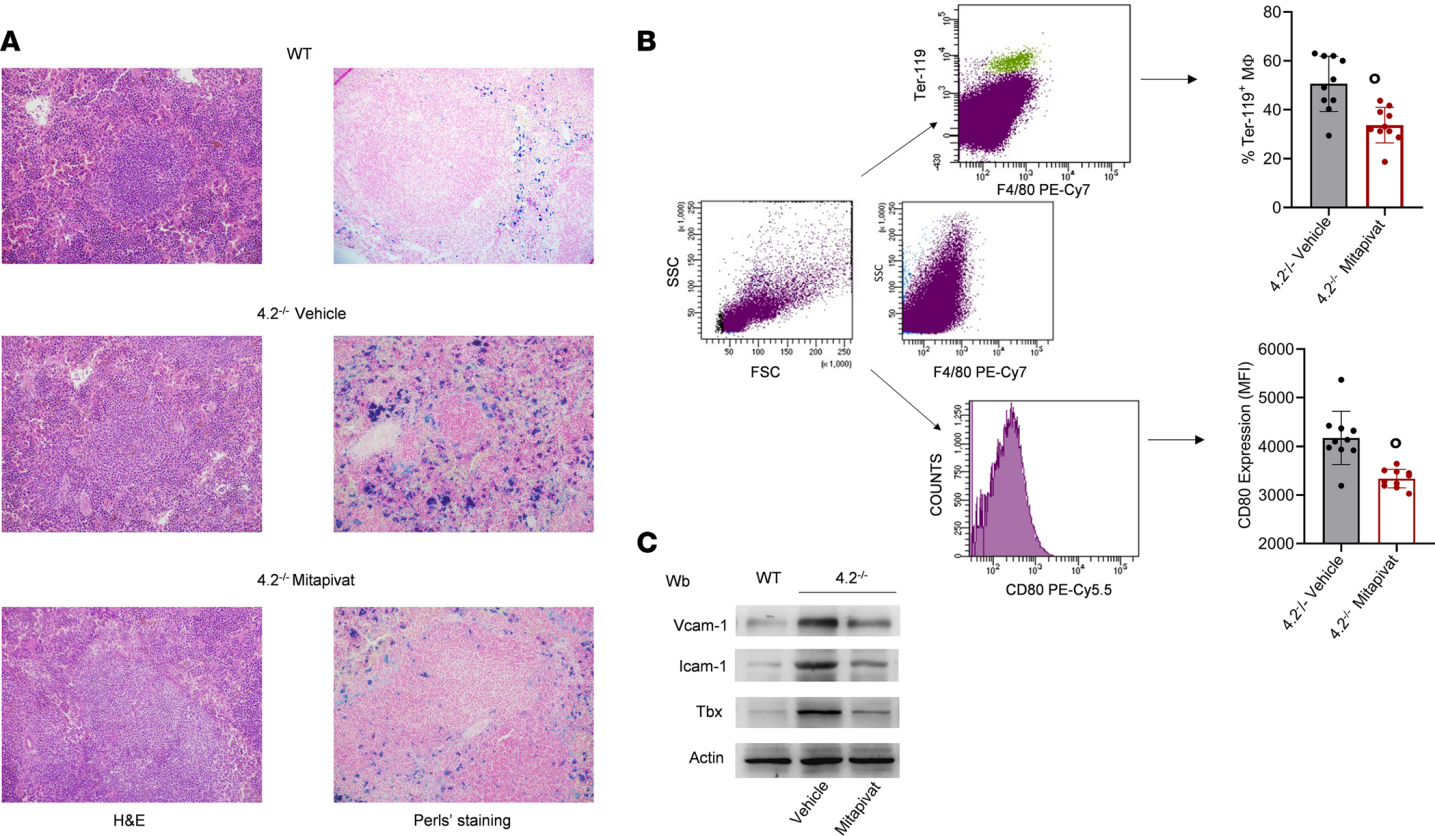
Mitapivat significantly reduces erythrophagocytosis, promotes a proresolving profile of splenic macrophages, and protects against hemolysis-induced inflammatory vasculopathy in 4.2^–/–^ mice. (**A**) H&E staining (left column) and iron staining (Perls’ Prussian blue; right column) in spleens from WT and 4.2^–/–^ mice treated with vehicle or mitapivat (100 mg/kg/d) for 6 months. Original magnification, ×200. Scale bars: 100 μM. One representative image from 4 with similar results is shown. (**B**) Percentage of Ter-119/F4/80 double-positive splenic macrophages isolated from 4.2^–/–^ mice treated with vehicle or mitapivat (100 mg/kg/d) for 6 months, determined by flow cytometry. In parallel, surface expression of the M1-like marker CD80 was determined (see gating strategies for intracellular and surface staining analysis in left-side plots). Results are reported as mean ± SD from 10 mice/group. °*P* < 0.05 compared with vehicle-treated 4.2^–/–^ mice (unpaired *t* test). (**C**) IB analysis using specific Abs against Vcam1, Icam1, and Tbx in isolated aortas from WT and 4.2^–/–^ mice treated with vehicle or mitapivat (100 mg/kg/d) for 6 months. Protein (50 μg) loaded on an 8% T, 2.5%C polyacrylamide gel. Actin was the protein loading control. One representative gel from 4–5 with similar results is shown. Densitometric analysis of IBs is shown in Supplemental Figure 6B. FSC, forward scatter; SSC, side scatter; Wb, Western blot.

In HS, splenic conditioning plays a central role in generation of spherocytes, release of erythroid vesicles, and erythrophagocytosis. Erythrophagocytosis and splenic macrophage function also contribute to iron recycling (38, 39). A significantly higher level of erythrophagocytosis was noted in spleens of 4.2^–/–^ mice compared with WT animals at both 3 and 8 months of age (Supplemental Figure 7A). Mitapivat markedly reduced erythrophagocytosis in spleens of 4.2^–/–^ mice when compared with 4.2^–/–^ controls (Figure 4B). Mitapivat significantly diminished surface expression of the pro-inflammatory marker CD80 on splenic macrophages of 4.2^–/–^ mice compared with vehicle-treated 4.2^–/–^ mice (Figure 4B). This is indicative of skewing toward a proresolving macrophage phenotype because a reduction of CD80 is a distinctive mechanism of active resolution, in agreement with the reduction in erythrophagocytosis (40).

Previous studies have shown that chronic hemolysis is associated with increased levels of markers of vascular activation and dysfunction in patients with different hereditary RBC disorders such as SCD, β-thalassemia, or HS (41), reflecting an increased risk of vascular complications (2, 33, 41, 42). As shown in Figure 4C, isolated aorta from 4.2^–/–^ mice displayed upregulation of Vcam1, Icam1, and thromboxane synthase (Tbx) that are involved in vascular activation, cell adhesion, and platelet activation (43). All these responses were blunted in mitapivat-treated 4.2^–/–^ mice (Figure 4C and Supplemental Figure 6B).

Collectively, these data support a role for mitapivat in reprograming macrophages from a pro-inflammatory to a proresolving state leading to a significant reduction in erythrophagocytosis in 4.2^–/–^ mice. In combination with the reduction in erythroid vesicle release, these changes can account for amelioration of inflammatory vasculopathy complications of HS.

### Mitapivat reduces liver iron overload and modulates c-duodenal Dmt1 expression in 4. 2^–/–^ mice.

**Figure 5:**
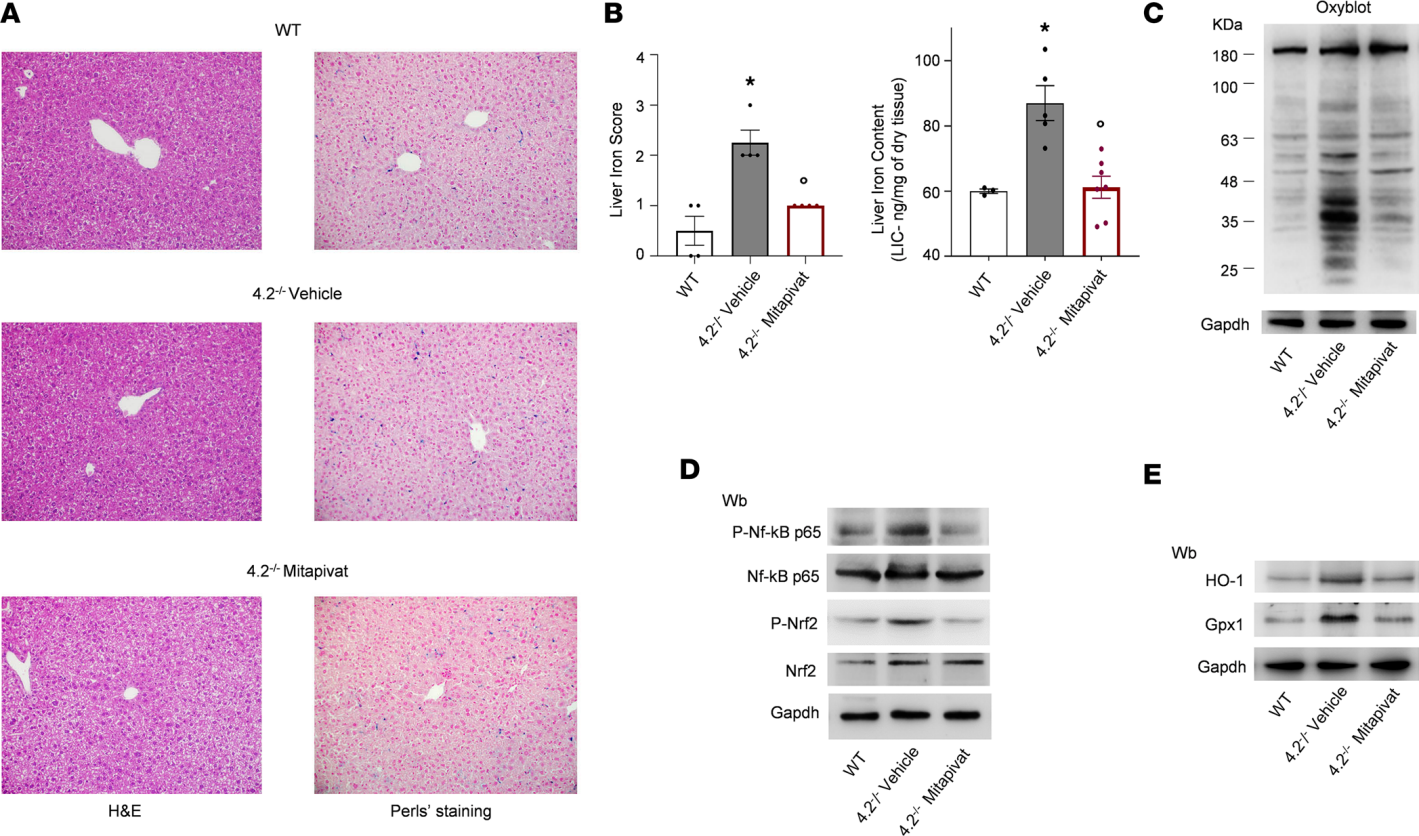
Mitapivat-treated 4.2^–/–^ mice had reduced liver iron accumulation and protection against liver oxidation. (**A**) H&E staining (left column) and iron staining (Perls’ Prussian blue; right column) in liver tissue from WT and 4.2^–/–^ mice treated with either vehicle or mitapivat (100 mg/kg/d) for 6 months. Original magnification, ×200. Scale bars: 100 μM. One representative image from 4 with similar results is shown. (**B**) Quantification of Perls’ iron staining of liver tissue (left bar graph) and the non-heme liver iron content determined using the bathophenanthroline staining method (right bar chart) in WT and 4.2^–/–^ mice treated with vehicle or mitapivat (100 mg/kg/d) for 6 months. Data are reported as mean ± SEM (*n* = 3–7). *P < 0.05 compared with WT mice and °*P* < 0.05 compared with vehicle-treated mice by 1-way ANOVA. (**C**) OxyBlot analysis of the soluble fractions of liver from WT and 4.2^–/–^ mice treated as in **A**. The carbonylated proteins (1 mg) were detected by treating with 2,4-dinitrophenylhydrazine and blotted with anti–DNP Ab. Gapdh was the protein loading control. Quantification of band area is shown in Supplemental Figure 7B. (**D**) IB analysis using specific Abs against phosphorylated (p-)NF-κB p65, NF-κB p65, (p-)Nrf2, and Nrf2 in liver tissue from WT and 4.2^–/–^ mice treated as in **A**. Protein (75 μg) was loaded on an 8% T, 2.5%C polyacrylamide gel. Gapdh was the protein loading control. One representative gel from 4 with similar results is shown. Densitometric analysis of IBs is shown in Supplemental Figure 7C. (**E**) IB analysis using specific Abs against HO-1 and Gpx1 in liver from WT and 4.2^–/–^ mice treated as in **A**. Protein (50 μg) loaded on an 11% T, 2.5%C polyacrylamide gel. Gapdh was the protein loading control. One representative gel from 4 with similar results is shown. Densitometric analysis of IBs is shown in Supplemental Figure 7D. Wb, Western blot.

Liver iron overload is a common and severe pathological feature of HS, a consequence of chronic hemolysis and inappropriate hepcidin downregulation (41, 44, 45). Mitapivat treatment of 4.2^–/–^ mice significantly reduced liver iron overload as documented by both Perls’ staining and liver iron concentration (Figure 5, A and B). This reduction was associated with upregulation of *Hamp* via *Id1*, linked to the activation of the Smad pathway (Supplemental Figure 8A). In mitapivat-treated 4.2^–/–^ mice, we noted decreased protein oxidation in liver as determined by Oxyblot (Figure 5C and Supplemental Figure 8B), which was accompanied by blunted activation of Nrf2 and NF-κB p65 (Figure 5D and Supplemental Figure 8C) with resultant downregulation of Nrf2-related cytoprotectors such as heme-oxygenase-1 (HO-1) and Gpx1 (Figure 5E and Supplemental Figure 8D) (17, 46, 47). In 4.2 ^–/–^ mice, we further confirmed that mitapivat improved the duodenal oxidative or hypoxic intracellular environment resulting in a reduction of iron uptake due to downregulation of *Dmt1* expression, as evidenced by reduced (a) c-duodenum iron accumulation; (b) expression of stress-associated Pkm2; (c) expression of Hif 2α and of the NF-κB p65 active form; and (d) *Dmt1* iron response element (IRE) mRNA transcripts (Supplemental Figure 9).

These data imply a beneficial effect of mitapivat in iron homeostasis by modulation of *Dmt1* gene expression, which directly affects iron absorption and thereby decreases iron overload.

### Noninferiority of mitapivat treatment versus splenectomy in 4.2^–/–^ mice and additional benefit of mitapivat in splenectomized 4.2^–/–^ mice

**Figure 6:**
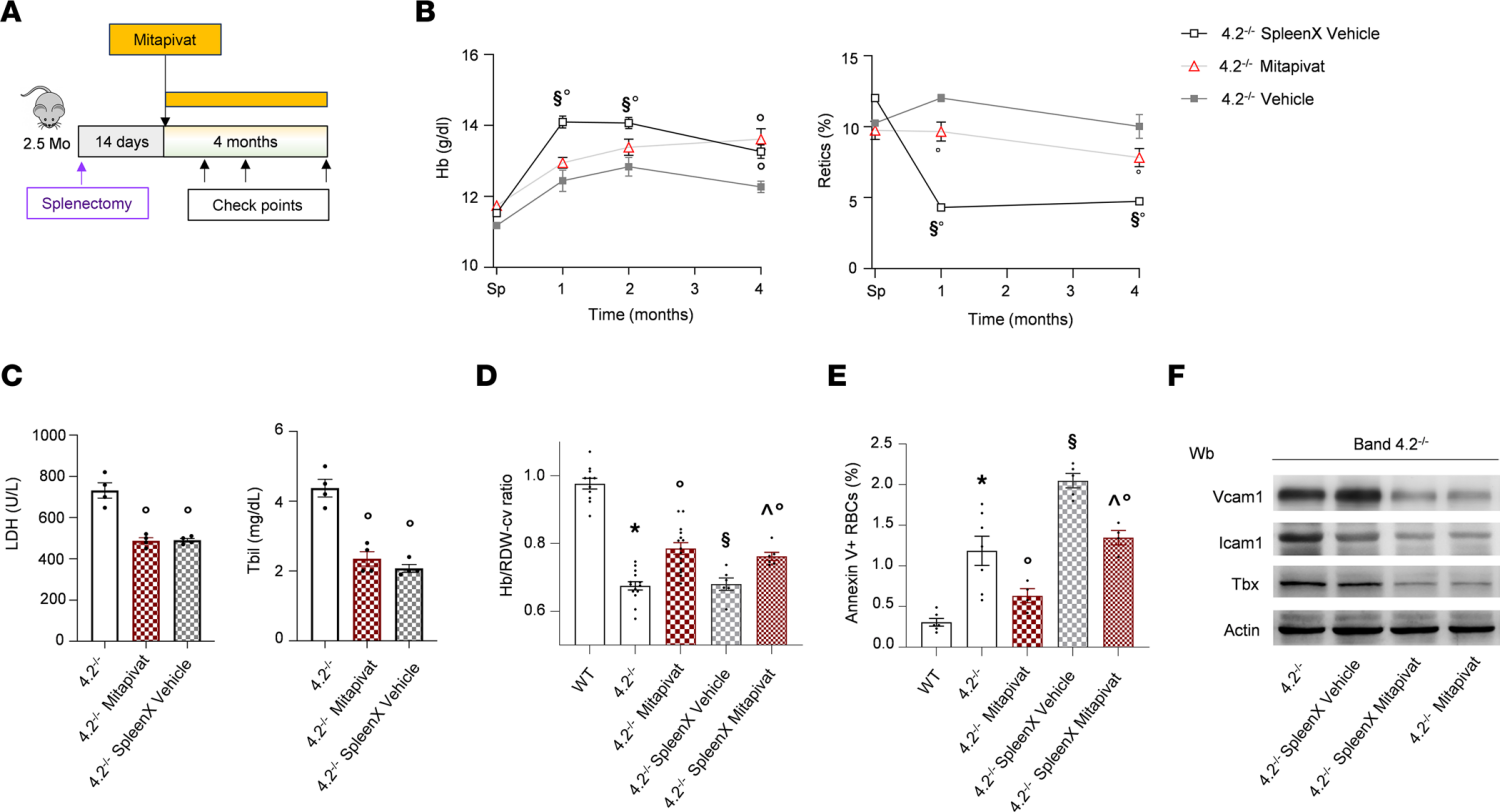
Noninferiority of mitapivat versus splenectomy in 4.2–/– mice is associated with protection against hemolysis-induced inflammatory vasculopathy. (**A**) Experimental design to study the effect of splenectomy in combination with mitapivat (100 mg/kg/d) in 4.2^–/–^ mice. (**B**) Hb levels and reticulocyte count in 4.2^–/–^ mice treated with vehicle or mitapivat (100 mg/kg/d) or splenectomized (spleenx) monitored up to 7 months of age. Data are reported as mean ± SEM; 4.2^–/–^ vehicle, *n* = 5–7; 4.2^–/–^ mitapivat, *n* = 5-7; 4.2^–/–^ spleenx, *n* = 6). °*P* < 0.05 compared with vehicle-treated 4.2^–/–^ mice; §*P* < 0.05 compared with mitapivat-treated 4.2^–/–^ mice (1-way ANOVA with Dunnett’s longitudinal comparison). (**C**) Plasma LDH and total bilirubin (Tbil) levels in 4.2^–/–^ mice treated as described in **A**. Data are reported as mean ± SEM (*n* = 4); °*P* < 0.05 compared with vehicle-treated 4.2^–/–^ mice (1-way ANOVA). (**D** and **E**) Hb to RDW ratio and annexin-V^+^ RBCs from WT and 4.2^–/–^ mice with or without spleen and treated with either vehicle or mitapivat (100 mg/kg/d). Data are reported as mean ± SEM (*n* = 4–15). *P < 0.05 compared with WT mice, °*P* < 0.05 compared with vehicle-treated 4.2^–/–^ mice; §*P* < 0.05 compared with mitapivat-treated 4.2^–/–^ mice; ^*P* < 0.05 compared with spleenx 4.2^–/–^ mice treated with vehicle (2-way ANOVA). (**F**) IB analysis using specific Abs against Vcam1, Icam1, and Tbx in isolated aortas from 4.2^–/–^ mice with or without spleen and treated with either vehicle or mitapivat. Protein (50 μg/μL) loaded on an 8% T, 2.5%C polyacrylamide gel. Actin was the protein loading control. One representative gel from 3–5 with similar results is shown. Densitometric analysis of IBs is shown in Supplemental Figure 9B. Wb, Western blot.

Splenectomy is the gold standard treatment for symptomatic HS. However, the indication for splenectomy in moderate to mild HS is still controversial and the cost-effectiveness of splenectomy is not well established. There is limited information on the benefits of splenectomy in murine HS; studies have been limited due to technical challenges and concerns that the spleen in mice is a site of both RBC destruction and production. We evaluated the impact of splenectomy in comparison with mitapivat treatment in 4.2^–/–^ mice (Figure 6A).

Splenectomy significantly increased Hb levels with a marked reduction in reticulocyte count when compared with control 4.2^–/–^ mice (Figure 6B). Mitapivat was noninferior to splenectomy for amelioration of anemia and reduction in reticulocyte count (Figure 6B), and both treatments resulted in similar decreases in hemolysis biomarkers such as LDH and total bilirubin (Figure 6C).

We further evaluated whether the administration of mitapivat might synergize with the beneficial effect of splenectomy in 4.2^–/–^ mice. Although the combined treatment did not further ameliorate Hb level and reticulocyte count (Supplemental Figure 10, A and B), it produced a greater change in the Hb to RDW ratio, a marker for spherocytes and dense RBCs (Figure 6D), and further decreased annexin-V positivity of RBCs (Figure 6E). Because PS-exposing cells are largely removed by spleen and can contribute to intravascular hemolysis and inflammatory vasculopathy, it is important to assess associated changes in vascular biomarkers (33, 41, 48). Splenectomized 4.2^–/–^ mice treated with mitapivat had significant reductions in Vcam1, Icam1, and Tbx expression in isolated aorta compared with splenectomized 4.2^–/–^ mice (Figure 6F and Supplemental Figure 10C). The effect of mitapivat alone or in combination with splenectomy on these markers of inflammatory vasculopathy was similar (Figure 6F), indicating that the mitapivat-induced reduction of hemolysis might be protective even in splenectomized 4.2^–/–^ mice.

## Discussion

HS is 1 of the most prevalent causes of hemolytic anemia due to membrane abnormalities (1, 2). The clinical management of HS is related to the severity of its hematologic phenotype and associated acute and chronic complications that include organ damage due to iron overload (1, 2).

We show here that mitapivat, an oral PK activator, ameliorates anemia in 4.2^–/–^ mice, a model for human HS, by reprograming the RBC metabolome. Indeed, mitapivat restores a more physiological flow of the glycolytic intermediates, enhancing the production of ATP (Supplemental Figure 3C) (16, 17, 49). This provides the critical element for the phosphorylation of glucose, which participates in GSH regeneration via production of NADPH in the pentose phosphate pathway, improving the RBC antioxidant response against physiologic stimuli such as the transit into the sluggish splenic circulation. In addition, mitapivat might favor the replenishment of RBC membrane ATP pools, which is important for membrane stability and ion transport function (10–12, 30, 31). This results in reduction of RBC vesiculation and band 3 loss with decreased hemolysis.

In 4.2^–/–^ mouse RBCs, the perturbation of the metabolome mainly involved the glycolytic pathway, a finding that is also reported in RBCs in HS and is associated with decreased membrane binding of glycolytic enzymes (7, 10). This condition affects the viability of ATP, which is compartmentalized into cytosolic and membrane-bound pools in RBCs (7, 11, 50). In addition, the membrane loss during splenic conditioning might further worsen the RBC metabolic setting, sustaining chronic hemolysis of HS. Abnormalities in glycolysis have been reported previously in human HS (4, 7). Similar to the findings reported by van Dooijeweert et al. (4) in human HS, RBC pyruvate content of 4.2^–/–^ mice was unchanged, but the PK activity was increased, most likely due to the persistent expression of both PKr and PKm2 isoforms and the increased reticulocyte count.

The changes in the metabolic profile of 4.2^–/–^ mouse erythrocytes induced by mitapivat treatment are not merely due to a reduction in reticulocyte counts. This conclusion is supported by the following findings: (a) the amelioration of RBC osmotic fragility; (b) the reduction in numbers of circulating annexin-V^+^ erythrocytes; and (c) the in vitro decrease generation of erythroid vesicles with normalization of their protein content. Mitapivat-induced changes are not limited to reticulocytes, as suggested by the observed significant reduction of erythrophagocytosis (Figure 4B) and the direct effect of mitapivat on splenic macrophages resulting in a proresolving profile. These findings are in accordance with our previous report of mitapivat treatment of β-thalassemic (Hbb^th3/+^) mice (39). Taken together, our data demonstrate for that mitapivat might reduce the splenic condition by targeting both RBCs and splenic macrophages.

The improvement of anemia in mitapivat-treated 4.2^–/–^ mice is also accompanied by reduction in liver iron overload. This reduction is due to upregulation of liver *Hamp* through the *Id1* pathway and associated downregulation of c-duodenal *Dmt1* expression, which is expected to result in diminished iron absorption.

In patients with HS, splenectomy is the gold standard therapeutic option for severe hematologic phenotype, but indications and long-term cost-effectiveness in moderate to mild HS are not well established (51). In the present study, we show the noninferiority of mitapivat versus splenectomy in 4.2^–/–^ mice with similar improvements in hematologic parameters and reduction in markers of hemolysis. The administration of mitapivat to splenectomized 4.2^–/–^ mice further improved the Hb to RDW ratio and reduced the amount of circulation PS^+^ RBCs, which, as expected, are increased by splenectomy.

Mitapivat-treated 4.2^–/–^ mice had a significant reduction in the expression of markers of inflammatory vasculopathy, including Vcam 1, Icam1, or Tbx. It was previously documented that in hereditary hemolytic anemias, splenectomy enhances intravascular hemolysis, aggravating the inflammatory vasculopathy and increasing the risk of thrombotic events (41, 51). Importantly, we show here that mitapivat protects against hemolysis induced inflammatory vasculopathy in 4.2^–/–^ mice with or without spleen.

In conclusion, mitapivat treatment of 4.2^–/–^ mice metabolically re-programs RBCs, ameliorates pathophysiology, and improves anemia. Important contributors to reduced hemolysis are reduced splenic conditioning with decreased erythroid vesicles release, reduced erythrophagocytosis, and a proresolving profile of splenic macrophages. In 4.2^–/–^ mice, we confirmed that mitapivat improves iron homeostasis by both reduction in chronic hemolysis and in c-duodenal iron absorption. Finally, we demonstrate the noninferiority of mitapivat versus splenectomy, generating the rationale for mitapivat as a potential alternative to splenectomy in mild-to-moderate HS. The reduction in markers of inflammatory vasculopathy observed in 4.2^–/–^ mice with or without spleen further corroborate the attractive therapeutic profile of mitapivat in HS and in other membranopathies such as stomatocytosis, where splenectomy is contraindicated due to a significantly increased thrombotic risk.

## Methods

### Mouse strains and study design.

Female WT C57B6 and 4.2^–/–^ (Epb42^tm1Llp^/LlpJ) mice were studied. Whenever indicated, 2-month-old mice were fed with standard diet enriched with 1200 ppm mitapivat (corresponding to a dose of 100 mg/kg/d) for up to 6 months (17). Blood was collected by retro-orbital venipuncture in anesthetized mice using heparinized microcapillary tubes (52, 53). Hematological parameters, RBC indices, and reticulocyte count were evaluated at baseline and at different time points, as previously reported, on a Sysmex XN-1000 Hematology Analyzer (Sysmex Corporation) (43). Hct and Hb values were manually determined (54).

Flow cytometric analysis of erythroid precursors from BM and spleen from WT and 4.2^–/–^ mice was carried out using the CD44-Ter119-Fsc^hi^ gating strategy (17). Total bilirubin and LDH levels were evaluated using standard biochemical assays, as previously reported (37). The concentration of mouse EPO in plasma was determined using the Mouse Erythropoietin Quantikine ELISA Kit (R&D Systems), following the manufacturer instructions. PK activity measurements were performed as described (17). ATP content was determined on washed erythrocytes using the CellTiter-Glo Luminescent cell viability assay (Promega Corp.), as previously reported (55). RBC Na^+^ and K^+^ content, as well as Na/K/Cl cotransport and Na/H exchange activities, were carried out as previously reported (5, 30, 31).

### Metabolomic analysis of RBCs.

Metabolomic analysis of RBCs was carried out as described by Gevi et al. (56, 57).

### Measurements of RBC osmotic fragility and erythroid vesicles.

RBC osmotic fragility and erythroid vesicles were determined by flow cytometric analysis as previously described (36, 58). Details are reported in Supplemental Methods.

### Molecular analysis of liver and duodenum.

Protocols used for RNA isolation, cDNA preparation, and qRT-PCR have been described (17). qRT-PCR was performed using the SYBR-green method as detailed in the Supplemental Methods; all primer sequences are listed in Supplemental Table 1. Western blots were performed as previously reported (17). Details are reported in Supplemental Methods. Immediately following dissection, the liver and duodenum were formalin fixed and paraffin embedded for Perls’ staining (39). Liver and spleen iron content was determined as previously described (17). Details are reported in Supplemental Data.

### Analysis of erythrophagocytosis and macrophage receptors.

Spleens were gently dissociated into single cells using GentleMACS dissociator (Miltenyi Biotec) and stained with F4/80 PE-Cy7 (Biolegend). Following staining, cells were fixed, permeabilized, and counterlabeled with anti–Ter-119 FITC (BioLegend) to measure macrophage intracellular fluorescence associated with phagocytosed RBCs. Cells stained as described above without permeabilization served as negative controls of intracellular staining, as previously reported (37). Anti–80 PerCP-Cy5.5 (BioLegend) was used to determine surface expression of phagocytic receptors on spleen and lung macrophages identified using an anti–F4/80 PE-Cy7 Ab. Flow cytometry was carried out on a BD FACS Canto II flow cytometer (BD Biosciences) and results were analyzed with the FACS DIVA software (BD Biosciences).

### Statistics.

Normality was assessed with the Shapiro-Wilk test. Data were analyzed using either a 1-tailed *t* test or 1-way or 2-way ANOVA. *P* values of less than 0.05 were considered significant. Details related to the statistical tests used are reported in each figure legend.

### Study approval.

The Institutional Animal Experimental Committee of University of Verona and the Italian Ministry of Health approved the experimental protocols (56DC9.64), following European directive 2010/63/EU and the Federation for Laboratory Animal Science Associations guidelines and recommendations.

### Data availability.

All the data are given in the Supplemental Data file. All the protocols are stored in the Nas Synology DS216se hard disk, located at the University of Verona, 37134 Verona, Italy, and are available on request.

## Author contributions

AM, EF, LDF, CB, NM, and AI designed the study; LDF, CB, NM, and AI wrote and revised the paper; AM, EF, and LDF carried out hematologic analysis, flow cytometric analyses, and IB studies, and analyzed data; LD discussed the data; ABW carried out the hematologic analysis; FG and AMT carried out and evaluated the metabolomic analyses; EG and GF analyzed spleen macrophages and erythrophagocytosis; AMB and MAP carried out the PK analysis; EG reviewed the paper; RR carried out the molecular analysis and contributed to writing the paper; CL and AJ performed histopathologic analysis. AR contributed in macrophage analysis and in writing the paper.

## Supplementary Material

Supplemental data

Supporting data values

## Figures and Tables

**Figure 7:**
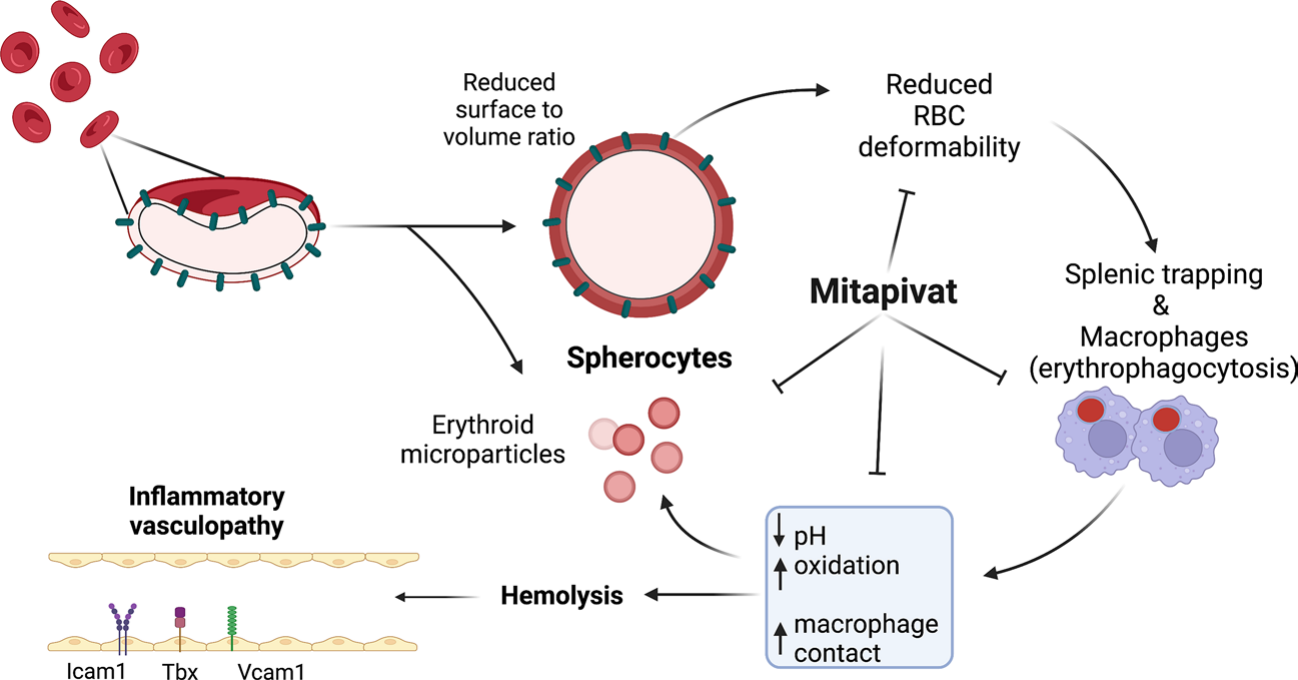
A proposed mode of action of mitapivat in murine HS. In 4.2^–/–^ mice, a model of HS, mitapivat improves anemia by metabolic reprograming of HS erythrocytes. This results in amelioration of HS RBC features with reduction of erythrophagocytosis and modulation of splenic macrophages toward a proresolving pattern with decreased splenic conditioning and reduced erythroid vesicle release. In 4.2^–/–^ mice, mitapivat induced improvement of chronic hemolysis reduces liver iron overload, modulates c-duodenum iron absorption via *Dmt1* downregulation, and protects against hemolysis-induced inflammatory vasculopathy.

## Version 1 09/07/2023

In-Press Preview

## Version 2 10/23/2023

Electronic publication
