# Supplementary material for: Mitapivat reprograms the RBC metabolome and improves anemia in a mouse model of hereditary spherocytosis
Source: JCI Insight. 2023 Oct 23;8(20):e172656. doi: 10.1172/jci.insight.172656 (PMC10619498; doi:10.1172/jci.insight.172656)
Supplement: Supplemental data [file jciinsight-8-172656-s272.pdf]

SUPPLEMENTAL DATA

METHODS

Drugs and chemicals

NaCl, Na_3VO_4, bicine, Tris, Tween 20, choline, $MgCl_2$, glycine, MOPS, benzamidine, β-mercaptoethanol, SDS, NaF, EDTA, May-Grünwald stain, Giemsa stain, BSA, glycerol, Luminata Forte and Luminata Classico HRP solutions, and dithiothreitol were obtained from Merck KGaA; protease inhibitor cocktail tablets were from Roche; Triton X-100, prestained Protein Ladder, and TEMED were from GE Healthcare; 40% acrylamide/bisacrylamide solution, 37.5:1 was from Bio-Rad; Dulbecco's PBS was from Lonza.

Mouse studies

Where mitapivat was administered by addition to the mouse diet, 1,200 ppm w/w was added to the Mod LabDiet 5P00 standard rodent diet, which corresponds to the oral 50 mg/kg twice a day dosage of mitapivat, as previously reported (17).

Red cell Western-blot analysis

Red cells were analyzed either in unfractionated form or after separation on a discontinuous Percoll density gradient that yielded fraction 1 (F1) corresponding to a density of N1.074 (containing reticulocyte enriched red cell fraction) and fraction 2 (F2) corresponding to a density of N1.092 as previously described (17).

Metabolomic analysis of red cells

100 µL of RBC was added to 1000 µl of a chloroform/methanol/water (1:3:1 ratio) solvent mixture stored at −20 °C. The tubes were mixed for 30 min and subsequently centrifuged at 1000×g for 1 min at 4 °C, before being transferred to −20 °C for 2–8 h. The solutions were then centrifuged for 15 min at 15,000×g and dried to obtain visible pellets. Finally, the dried samples were resuspended in 0.1 mL of water, 5% formic acid and transferred to glass autosampler vials for LC/MS analysis. Twenty-microliter of extracted supernatant samples was injected into an ultra-high-performance liquid chromatography (UHPLC) system (Ultimate 3000, Thermo) and run in positive ion mode. A Reprosil C18 column (2.0 mm × 150 mm, 2.5 µm - Dr Maisch, Germany) was used for metabolite separation. Chromatographic separations were achieved at a column temperature of 30 °C and flow rate of 0.2 mL/min. A 0 –100% linear gradient of solvent A (ddH2O, 0.1% formic acid) to B (acetonitrile, 0.1% formic acid) was employed over 20 min, returning to 100% A in 2

min and a 1-min post-time solvent A hold. The UHPLC system was coupled online with a mass spectrometer Q-Exactive (Thermo) scanning in full MS mode (2 μscans) at 70,000 resolution in the 60–1000 m/z range, target of 1 × 106 ions and a maximum ion injection time (IT) of 35 ms. Source ionization parameters were: spray voltage, 3.8 kV; capillary temperature, 300 °C; sheath gas, 40; auxiliary gas, 25; S-Lens level, 45. Calibration was performed before each analysis against positive ion mode calibration mixes (Piercenet, Thermo Fisher, Rockford, IL) to ensure sub ppm error of the intact mass. Data files were processed by MAVEN.8.1 (http://genomics-pubs.princeton.edu/mzroll/) upon conversion of raw files into mzXML format through MassMatrix (Cleveland, OH) and then processed by MAVEN 5.2 (available at http://genomics-pubs.princeton.edu/mzroll/); spectrometry chromatograms were assessed for peak alignment, matching and the comparison of parent and fragment ions, and tentative metabolite identification (within a 2 ppm mass-deviation range between observed and expected results against the imported KEGG database). Furthermore, t-test analysis of variance was performed using GraphPad Prism (version 9.0.0 (121), GraphPad Software, San Diego, CA, USA, www.graphpad.com) with $p \leq 0.05$ considered as being statistically significant. The results are expressed as mean ± standard deviation (SD) in the tables, and box and whisker plots were generated to show all points as well as median and ranges (whiskers = min. and max.) (47-48).

Pyruvate Kinase Assay

The pyruvate kinase activity was measured by using a commercial pyruvate kinase assay kit (Sigma-Aldrich, MAK072). Following the manufacturer's instructions, red blood cells were homogenized with pyruvate kinase assay buffer and spun at 15,000xg for 10 minutes to remove cell debris. Aliquots of the supernatant were transferred to a clear-bottom 96-well plate where the appropriate reaction mixtures were added to a final volume of 100 μl per well. All measurements were performed by measuring the absorbance of each sample at 570 nm using a TECAN Infinite M200 microplate reader (Tecan, Männedorf, Switzerland).

Measurements of red cells osmotic fragility and erythroid vesicles

Red cell osmotic fragility was evaluated by flow cytometric analysis as previously described (10). Briefly, heparinized blood was centrifuged, plasma and buffy coat were removed, RBCs were washed twice with PBS, 1% BSA (320 mOsm) and incubated with anti Ter119-APC (# 17-5921, eBiosciences, ThermoFisher Scientific).

Cells were centrifuged at 3000 rpm for 5 min and washed with PBS BSA 1%. The same number of RBCs were incubated in osmotic test solutions (NaCl solution at 192 mOsm) or isosmotic solution (320 mOsm) for 10 minutes at 37°C. Lysis was immediately stopped with 4x volumes of quenching solution (PBS at 320 mOsm) added of Count Bright Absolute Counting Beads (#C36950, eBiosciences, Thermo Fisher Scientific). Cells with normal flow cytometric FSC/SSC profiles were considered to be intact. Flow cytometric analysis was carried out with the FACSCanto I flow cytometer (Becton Dickinson). The biparametric scatter plots were analyzed with FlowJo software version 10 (Tree Star). The number of acquired counting beads was used to calculate the absolute number of intact red cells and percent of lysis was determined by the normalization to the 320 mOsm control condition (0% of lysis).
Erythroid vesicles were evaluated as previously described (23). Briefly, platelet pure plasma (PPP) was prepared from heparinized blood collected from wild-type and, $4.2^{-/-}$ mice treated with either vehicle or mitapivat (100 mg/Kg/day) for 5 months. Blood was centrifuged at 3000 rpm for 10 min. red cells were removed, and plasma was collected and centrifuged again at 3500 rpm for 15 min to obtain PPP. 5 µl of PPP were incubated in 50 µl Ringer buffer (32 mM Hepes-HCl pH 7.4, 125 mM NaCl, 5 mM glucose, 5 mM KCl, 1 mM $MgSO_4$, 2.5 mM $CaCl_2$), 0.2% BSA added of 0.5 µl of anti Ter119-APC ((# 17-5921-82, eBiosciences), 0.5 ul of AnnexinV-PE (# 128102-69, eBiosciences), 0.05 µl Phalloidin FITC (# F432, ThermoFisher Scientific, to remove RBC debris and ghosts). After 30 min of incubation at 4°C wash with 100 µl ringer buffer, 0.2% BSA added of Count Bright Absolute Counting Beads. Flow cytometric analysis was carried out with the FACSCanto I flow cytometer (Becton Dickinson). The biparametric scatter plots were analyzed with FlowJo software version 10 (Tree Star). The amounts of plasmatic vesicles was determined using the formula:

$$Absolute\ count\ \left(\frac{cells}{\mu L}\right) = \frac{(Cell\ count\ \times Counting\ beads\ volume)}{(Counting\ beads\ count\ \times Cell\ volume)} \times Counting\ beads\ concentration\ (\frac{beads}{\mu L})$$

Analysis of in vitro release of erythroid vesicles was carried out as previously reported (Ferru E et al. Blood 2011). Briefly, red cells from wild-type (WT) and $4.2^{-/-}$ mice were washed 3 times with PBS, 5 mM Glucose and incubated, at 3% Hct, in preservation buffer (155 mM KCl, 1 mM NaCl, 0.25 mM KPO4 pH 7.4, 1 mM

glucose) added of vehicle or mitapivat (2 μM) for 1h at 37°C. After 3 washes with PBS, 5 mM glucose at 4°C, treated red cells were incubated under shacking conditions, 1200 rpm, to mimic *in vivo* share stress at 42°C, for 50 min in presence of vehicle or mitapivat (2 μM). The suspension was then centrifuged at 3000 rpm 5 min at 4°C, RBC were removed, and the supernatant was used either for flow cytometric analysis of erythroid microparticles or centrifuged at 13000 rpm 5 min to remove ghosts, ultracentrifuged at 100,000 g for 2 h, to collect MP for western blot analysis. The following antibodies were used: anti Band-3 (Clone IVF12, Developmental Studies Hybridoma Bank, Iowa City, IA, USA, dilution 1:1000) and anti-peroxiredoxin-2 C-terminal (kindly gift of Prof. Chae HZ, Chonnam National University, South Korea). Coomassie staining was used to visualize the general protein pattern of erythroid vesicles.

Flow cytometric analysis of mouse erythroid precursors

Briefly, 500,000 cells from bone marrow were incubated with the following antibodies from eBiosciences (ThermoFisher Scientific): anti-CD16/CD32 blocking agent (clone 93), anti-CD44-FITC (clone IM7), anti-CD71-PE (clone R17217), anti-Ter119-APC(# 17-5921-82); anti-CD45 APC-eFluor 780 (clone 30F-11) for 30 minutes at 4°C. Flow cytometric analysis was carried out with the FACSCanto I flow cytometer (Becton Dickinson). The biparametric scatter plots were analyzed with FlowJo software version 10 (Tree Star) (17). Total amount of erythropoietic cells and erythroid populations were analysed according to the CD44 Ter119 Fsc-A gating strategy (Fig 4Sc).

Liver and duodenum molecular analysis

Western-blot analysis. Frozen livers from WT and $Hbb^{th3/+}$ mice were homogenized and lysed with ice cold lysis buffer (150 mM NaCl, 25 mM bicine, 0.1% SDS, 2% Triton X-100, 1 mM EDTA, protease inhibitor cocktail tablets, 1 mM Na_3VO_4 final concentration) followed by centrifugation for 30 minutes at 4°C at 12,000*g*. Proteins were quantified then separated by monodimensional SDS-PAGE. Proteins were transferred to nitrocellulose membranes for western blot analysis with specific antibodies: PKLR (dilution 1:1000, 75 μgr loaded, AbCam, Cambridge, UK), PKM2 (# 4053, Cell Signaling Technology) (dilution 1:1000, 75 μgr loaded), Catalase (dilution 1:1000, 75 μgr loaded, AbCam, Cambridge, UK), Band3 IVF12 (Developmental Studies Hybridoma Bank, DSHB, University of Iowa, USA) (1:1000 dilution, 20 μgr loaded), PRX 2 (kindly provided by Prof. Ho Zoo Chae, School of

Biological Science and Technology, Chonnam National University, Gwangju, Korea), VCAM-1 (R and D Systems, Minneapolis, MN, USA) (dilution 1:1000, 40 µgr loaded); ICAM-1 (clone EP1442Y, dilution 1:1000, 75 µgr loaded, AbCam, Cambridge, UK); TXAS-1 (Cayman, Ann Arbor, MI, USA) (dilution 1:1000, 75 µgr loaded), Phospho-Ser536 NF-κb p65 (pNF-κb p65, # 3031, Cell Signaling Technology) (dilution 1:1000, 75 µgr loaded), NF-κb p65(clone C22B4, Cell Signaling Technology) (dilution 1:1000, 75 µgr loaded), Phospho- Ser40 NRF2 (pNRF2, clone EP1809Y, AbCam) (dilution 1:1000, 75 µgr loaded), and NRF2 (clone EP1809Y, Abcam) (dilution 1:1000, 75 µgr loaded), anti-heme oxygenase 1 (HO-1, dilution 1:1000, 50 µgr/µl loaded, SCBT Santa Cruz, CA, USA), Gpx1 (dilution 1:1000, 50 µgr/µl loaded, clone N-20, Santa Cruz Biotechnology, CA, USA), HIF2α (clone 190b; Santa Cruz Biotechnology) (dilution 1:1000, 75 µgr loaded). Anti-GAPDH (clone D6, Santa Cruz Biotechnology, CA, USA) and anti-Actin (Santa Cruz Biotechnology, CA, USA (clone 2A3, dilution 1:1000, 50 µgr loaded) were used as loading control. Secondary donkey anti-rabbit IgG (dilution 1:10000) and anti-mouse IgG (dilution 1:5000) HRP conjugates were from GE Healthcare Life Sciences (Little Chalfont, UK), secondary donkey anti-goat (dilution 1:10000) HRP conjugate was from Santa Cruz Biotechnology. Blots were developed using the Luminata Forte Chemiluminescent HRP Substrate from Merck KGaA, and images were acquired with the Alliance Q9 Advanced imaging system (Uvitec, UK). Oxidized proteins were monitored using OxyBlot Protein Oxidation Detection Kit (EMD Millipore) as previously reported (4), briefly, carbonylated proteins were detected by treating with 2,4-dinitrophenylhydrazine (DNPH) and blotted with anti- dinitrophenyl antibody. Densitometric analyses were performed with the Nine Alliance software (Uvitec, UK) (17).

RT-PCR analysis. Total RNA was extracted from mouse tissues (liver and C-duodenum) using TRIzol reagent (Life Technologies). Synthesis of cDNA from total RNA (2 µg) was performed using SuperScript II First Strand kits (Life Technologies) and qRT-PCR was performed with SYBR Green PCR Master Mix (Applied Biosystems) using Applied Biosystems Model 7900HT Sequence Detection System. Detailed primer sequences are available in Supplemental Table 1. All PCR reactions were performed in triplicate. Relative gene expression was calculated using the $2^{-\Delta Ct}$ method, in which Ct indicates cycle threshold, the cycle number where the fluorescent signal reaches the detection threshold. ΔCt was computed by calculating

the difference of the average Ct between the test gene and the internal control gene, *Gapdh* (17).

Liver and spleen iron content. Spleen and liver samples were dried at 90°C overnight and weighed. About 10-20 mg of dry tissue was digested in 1 mL of acid solution (3 M HCl, 0.6 M trichloroacetic acid) at 65°C for 16 hours. Next, 20 µL of the acid extract were analyzed using 1 mL of chromogen solution (0.1% bathophenanthroline sulfate, 1% idrossilammine, and 10% sodium acetate pH 6.5), evaluating the absorbance at 535 nm. A standard curve was generated using an acid solution containing increasing amounts of iron sulfate (17).

Analysis of erythrophagocytosis and macrophage receptors

Spleens were gently dissociated into single cells using GentleMACS dissociator (Miltenyi Biotec, Germany) and stained with F4/80 PE-Cy7 (Biolegend, CA, United States). Following staining cells were fixed, permeabilized, and counter-labeled with anti-Ter-119 FITC (Biolegend) to measure macrophage intracellular fluorescence associated with phagocytosed red cells. Cells stained as above without permeabilization served as negative controls of intracellular staining, as previously reported (24). Anti-CD80 PerCP-Cy5.5 (Biolegend) was used to determine surface expression of phagocytic receptors on spleen and lungs macrophages identified using an anti-F4/80 PE-Cy7 antibody.

Flow cytometry was carried out on a BD FACS Canto II (BD Biosciences, NJ, United States) and results were analyzed with the FACS DIVA software (BD Biosciences).

Supplemental Table 1S. List of primers used in quantitative real-time PCR

Gene	Forward primer sequence (5' → 3')	Reverse primer sequence (5' → 3')
Hamp	GCCTGAGCAGCACCACCTAT	TTCTTCCCCGTGCAAAGGCT
Id1	CACTGAGGGACCAGATGGACTC	GGTGGCTGCGGTAGTGTCTT
Dmt1-IRE	GCAGTGTTTGATTGCATTGG	TCTTCGCTCAGCAGGACTTT
Dmt1	TCTTCTGAACACCGTGGATG	CCGAAAGGGCTTAGAGAAAG
Gapdh	CCACATCGCTCAGACACCAT	AGTTAAAAGCAGCCCTGGTGAC

SUPPLEMENTAL FIGURES

Figure 1S

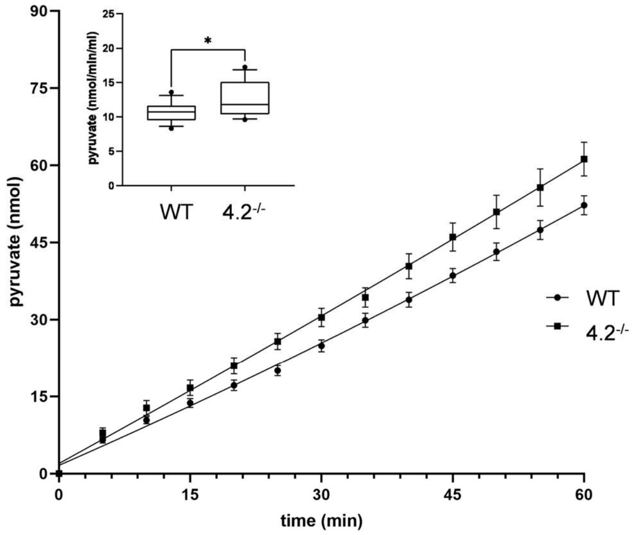

Figure 1S. Pyruvate kinase (PK) activity of red blood cell samples from wild-type (WT) and $4.2^{-/-}$ mice was recorded by measuring A_{570} every 5 minutes for 60 minutes. The absorbance values were converted to nmol of pyruvate produced by one microliter of red cell lysate based on the standard curve generated by serial dilutions of the pyruvate standard solution following the instructions of the manufacturer of the commercial kit (MAK072) and plotted as the means ± S.E.M. 4 experiments were performed in triplicate comparing the PK activity of WT mice with that of $4.2^{-/-}$ mice. The inset shows the PK activity recorded after 30 minutes' incubation and converted to nmol/min/ml following the manufacturer's instructions. The statistical significance of the experimental results was determined by unpaired t-test with Welch's correction *, $p < 0.05$.

Figure 2S

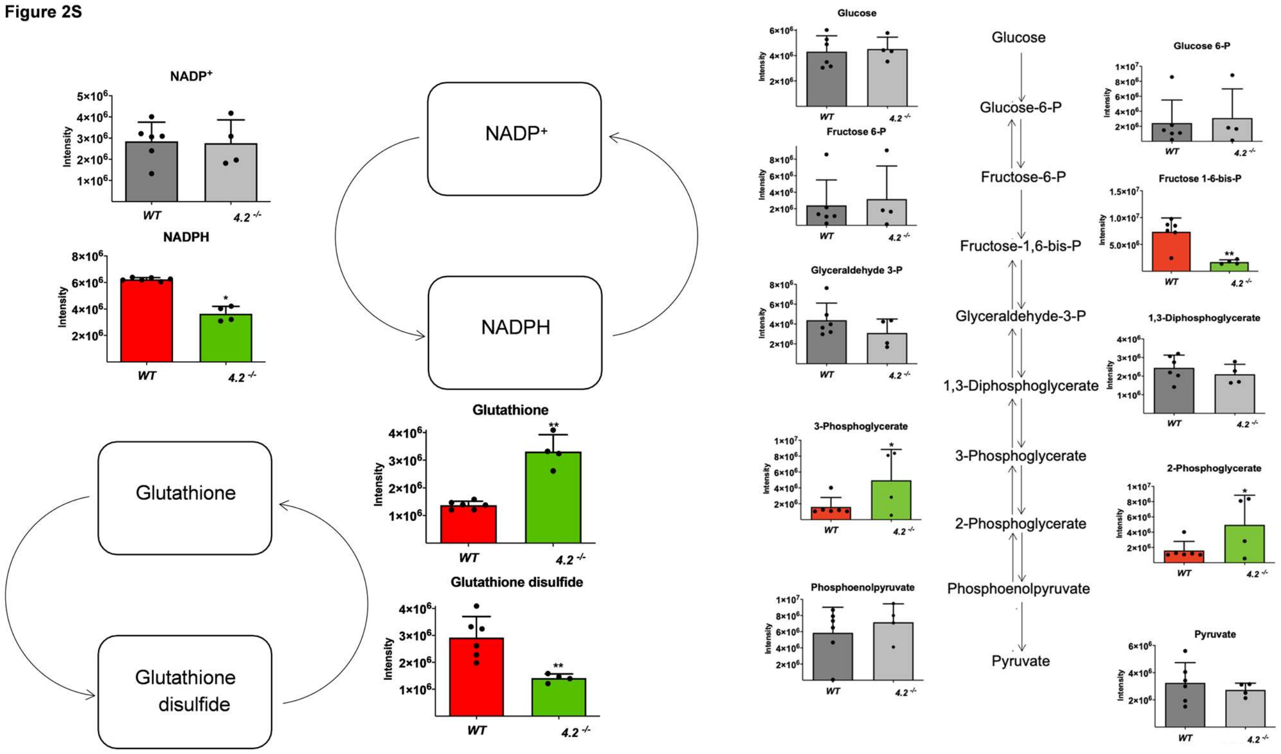

Figure 2S. Schematic representation of the NADPH/GSH (left panel) metabolism and glycolytic intermediates plotted for wild-type (WT) and $4.2^{-/-}$ red blood cells (RBCs). Data are means ± SD (n=4-6). * P < 0.05 compared to WT. ** P < 0.01 compared to WT by t-test.

Figure 3S

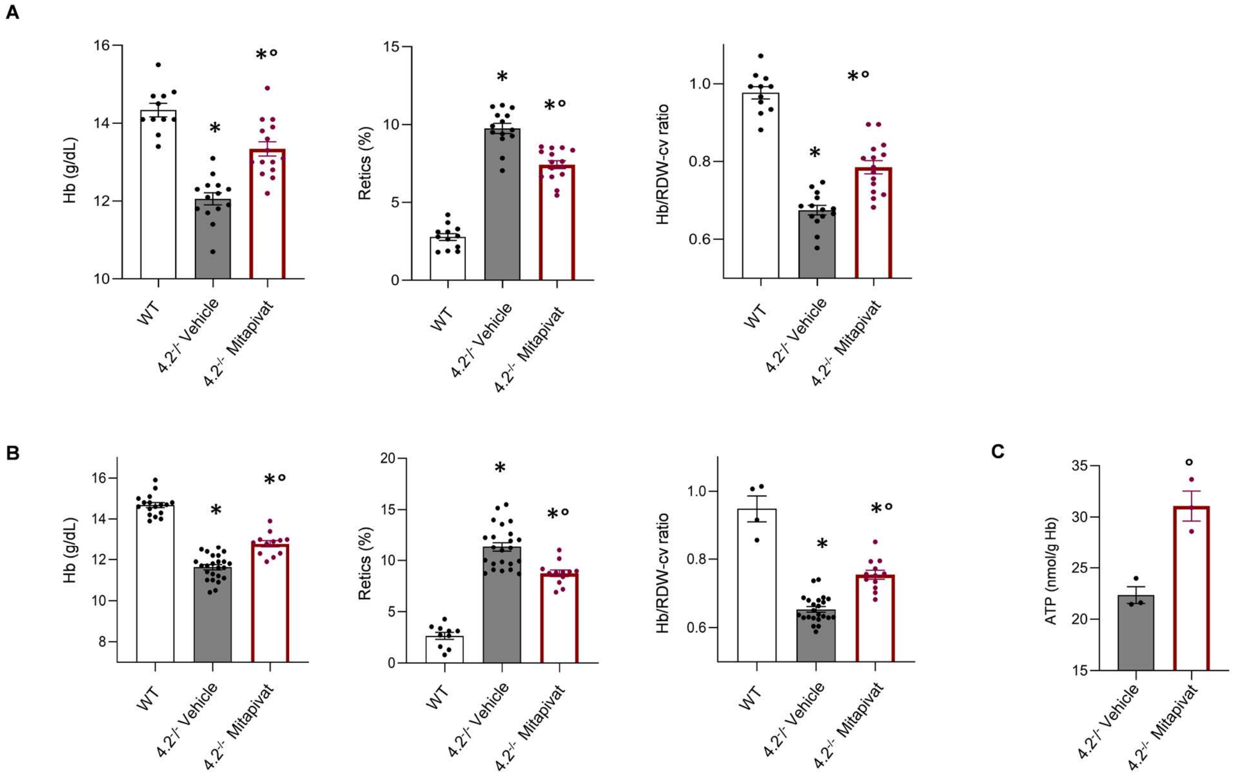

Figure 3S. Hemoglobin (Hb), Retics count and Hb/RDW ratio, as marker of spherocytosis, in WT and $4.2^{-/-}$ mice treated with vehicle or mitapivat for 5 **(A)** and 6 **(B)** months, starting at 2 months of age. Data are means ± SEM (n=4-23). * P < 0.05 compared to WT; ° P< 0.05 compared to vehicle treated $4.2^{-/-}$ mice by one way ANOVA. **(C)** ATP content in washed red cells from $4.2^{-/-}$ mice treated with vehicle or mitapivat. Data are presented as means ± SEM (n=3); ° P< 0.05 compared to vehicle treated $4.2^{-/-}$ mice by unpaired t-test with Welch's correction.

Figure 4S

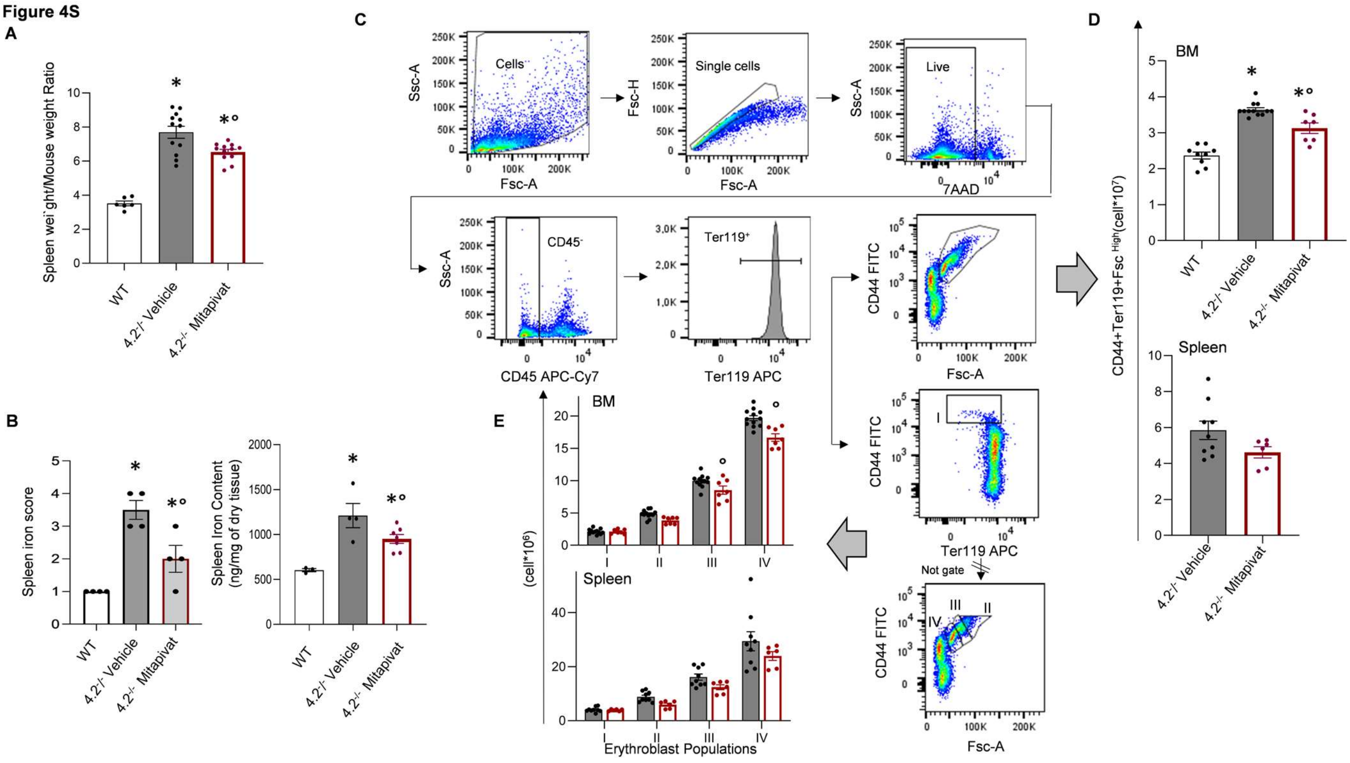

Figure 4S. A. Spleen weight:mouse weight ratio (mg/g) in wild-type (WT) and $4.2^{-/-}$ mice treated with vehicle or mitapivat (100 mg/Kg/day) for 6 months. Data are mean ± SEM (n = 6-12). * P < 0.05 compared with WT mice and ° P < 0.05 compared with vehicle-treated mice by one-way ANOVA. **B.** Quantification of the splenic Perl's iron staining (upper panel) and the non-Heme splenic iron content determined using the bathophenanthroline staining method (lower panel), in WT and $4.2^{-/-}$ mice treated with vehicle or mitapivat (100 mg/Kg/day) for 6 months. Data are mean ± SEM (n = 3-7). * P < 0.05 compared with WT mice and ° P < 0.05 compared with vehicle-treated mice by t-test. **C-E.** Representative scatter plots of the gating strategy used to analyze erythropoiesis and total amount of erythropoietic cells in the bone marrow (BM, **D**-upper panel bar graph) and spleen (**D**-lower panel bar graph) and in WT and $4.2^{-/-}$ mice treated with either vehicle or mitapivat (100 mg/Kg/day) for 6 months. Data are mean ± SEM (n = 6-12). * P < 0.05 compared with WT mice and ° P < 0.05 compared with vehicle-treated mice by one-way ANOVA. **E.** Erythroblast populations in bone marrow and spleen from $4.2^{-/-}$ mice treated with vehicle or mitapivat (100 mg/Kg/day) for 6 months. Population I (Pop I) corresponding to pro-erythroblasts, population II (Pop II), corresponding to basophilic erythroblasts; population III (Pop III), corresponding to polychromatic erythroblasts and population IV (Pop IV), corresponding to orthochromatic erythroblasts. Data are shown as mean ± SEM (n=6-12). ° P < 0.05 compared with vehicle-treated mice by two-way ANOVA.

Figure 5S

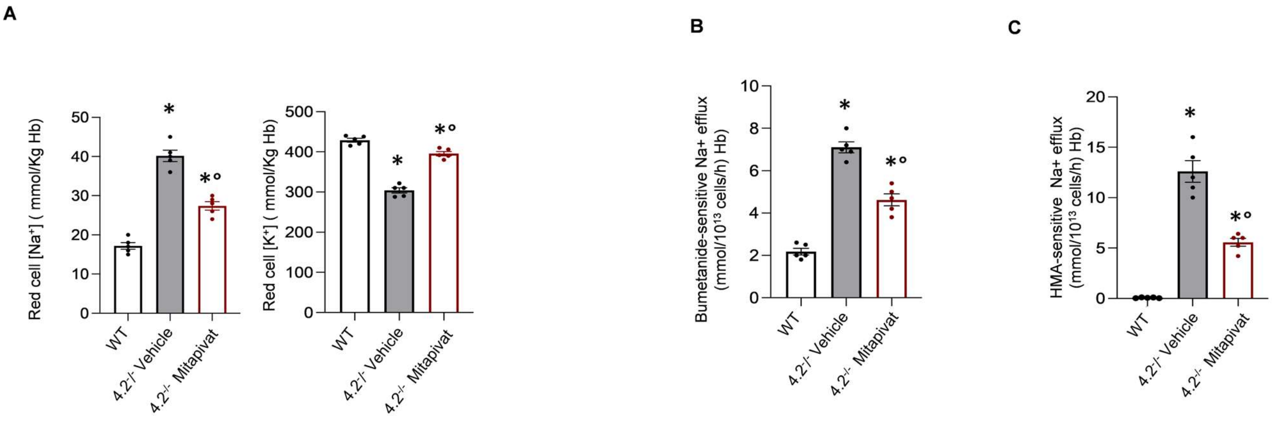

Figure 5S. A. Cation content (Na^+, K^+) in red cells from wild-type (WT) and $4.2^{-/-}$ mice treated with vehicle or mitapivat (100 mg/Kg/day) for 6 months. Data are mean ± SEM (n = 5). * $P < 0.05$ compared with WT mice and ° $P < 0.05$ compared with vehicle-treated mice by one-way ANOVA**. B.** Bumetanide (10 uM) sensitive Na/K/2Cl cotransport activity as Na+ efflux in red cells from wild-type (WT) and $4.2^{-/-}$ mice treated with vehicle or mitapivat. Data are mean ± SEM (n = 5). * $P < 0.05$ compared with WT mice and ° $P < 0.05$ compared with vehicle-treated mice by one-way ANOVA. **C.** Amiloride (10 uM) sensitive Na/H exchange activity as Na+ efflux in red cells from wild-type (WT) and $4.2^{-/-}$ mice treated with vehicle or mitapivat. Data are mean ± SEM (n = 5). * $P < 0.05$ compared with WT mice and ° $P < 0.05$ compared with vehicle-treated mice by one-way ANOVA.

Figure 6S

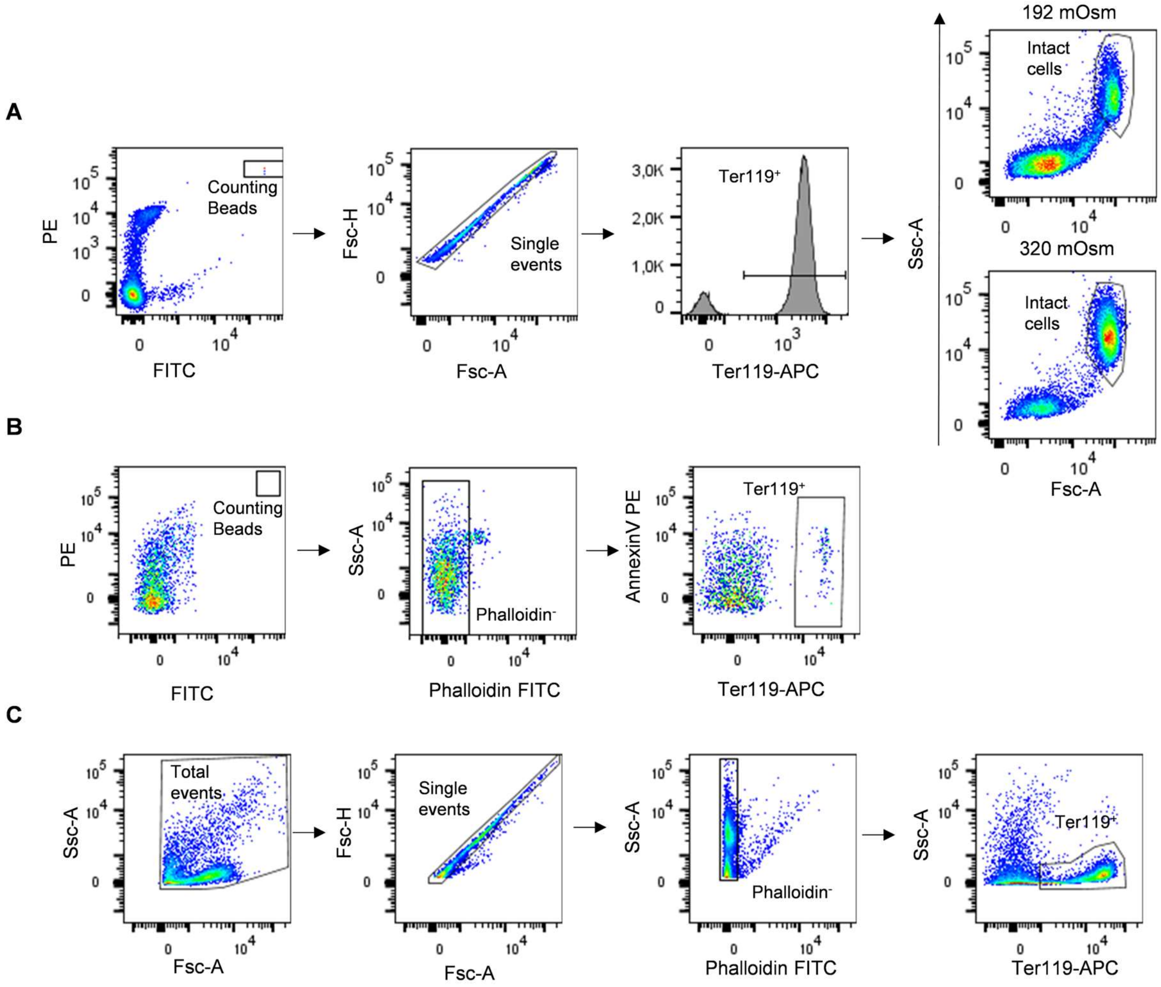

Figure 6S. Representative scatter plots of the gating strategy used to analyze **(A)** red blood cells (RBCs) osmotic fragility, **(B)** Erythroid plasmatic vesicles, **(C)** *in vitro* share stress induced erythroid vesicles.

Figure 7S

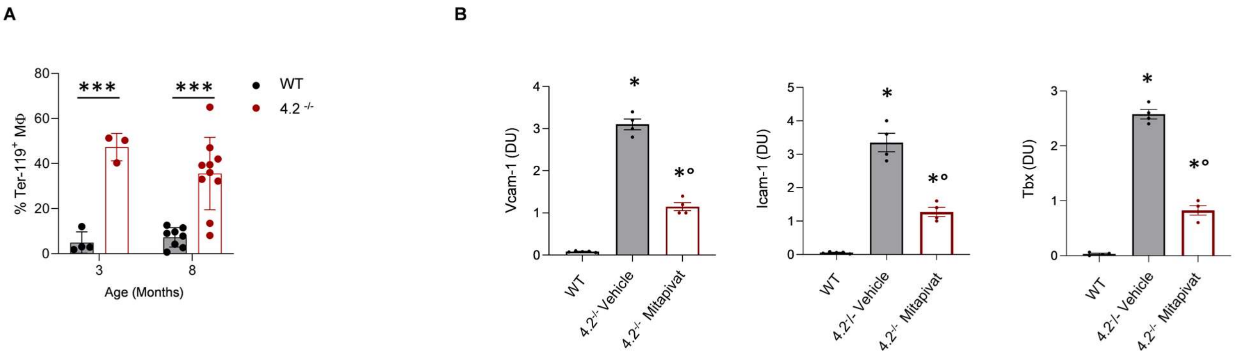

Figure 7S. A. Time course of Ter-119⁺ spleen macrophages in $4.2^{-/-}$ and wild-type (WT) mice. Percentage of Ter-119/F4/80 double positive splenic was determined with flow cytometry (see Materials and Methods) in macrophages isolated from wild-type (WT) or 4.2 $^{-/-}$ mice at indicated months of age. Results are mean ± SD from 3-4 (3 months-old mice) or 8-10 (8 months-old) mice/group. ***, P < 0.001 (Two-way ANOVA and Sidak post-hoc tests). **B.** Densitometric analysis of Western-blots as in Figure 4C. data are presented as mean ±SEM (n=4-5), * P < 0.05 compared with WT mice and ° P < 0.05 compared with vehicle-treated mice by t-test.

Figure 8S

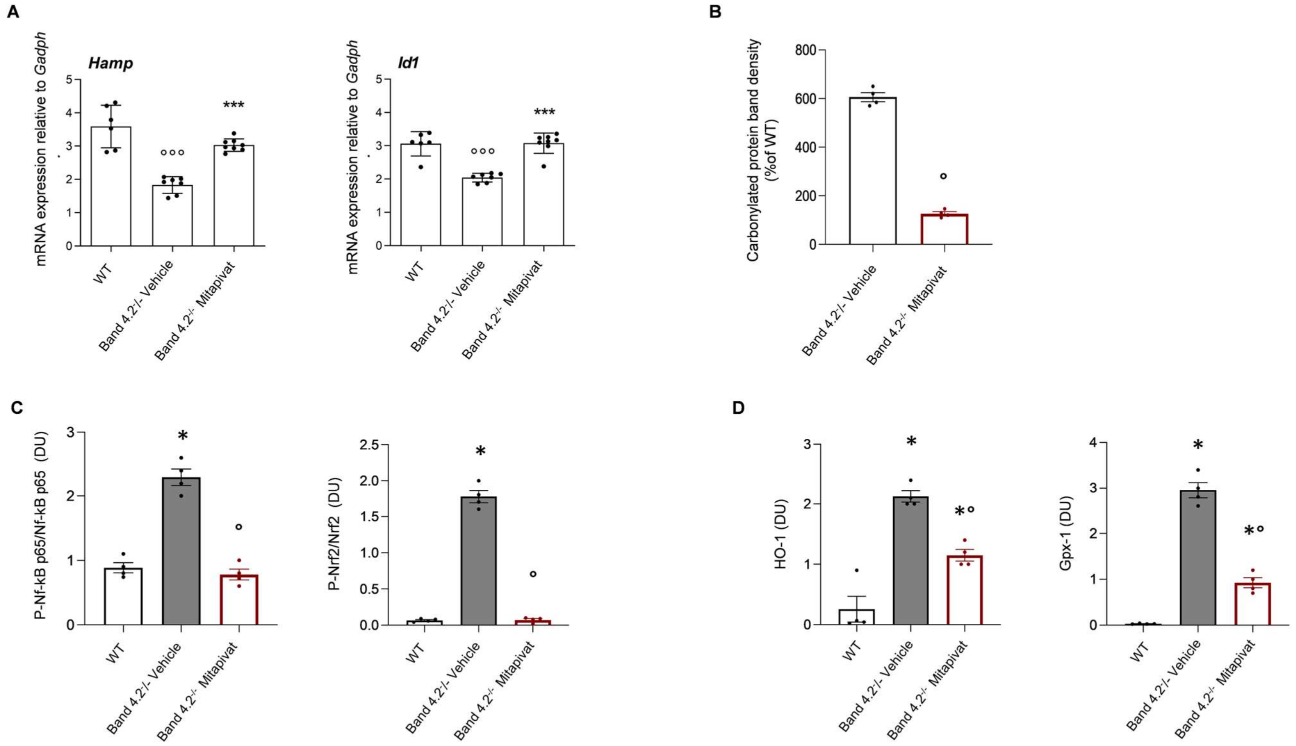

Figure 8S. A. mRNA expression of hepcidin (*Hamp*) and *Id1* by qRT-PCR on the liver from WT and $4.2^{-/-}$ mice treated with vehicle or mitapivat (100 mg/Kg/day) for 5 months. Experiments were performed in triplicate. Data are mean ± SD (n=6-8). °°°P < 0.01, $4.2^{-/-}$ vehicle vs WT mice; ***P < 0.01 vehicle vs mitapivat treated mice. P value was calculated by ANOVA, internal comparisons by post-hoc correction by Tukey's multiple comparisons test. **B-D.** Densitometric analysis of Western-blots as in Figure 5C-E. Data are presented as mean ±SEM (n=4), * P < 0.05 compared with WT mice and ° P < 0.05 compared with vehicle-treated mice by t-test.

Figure 9S

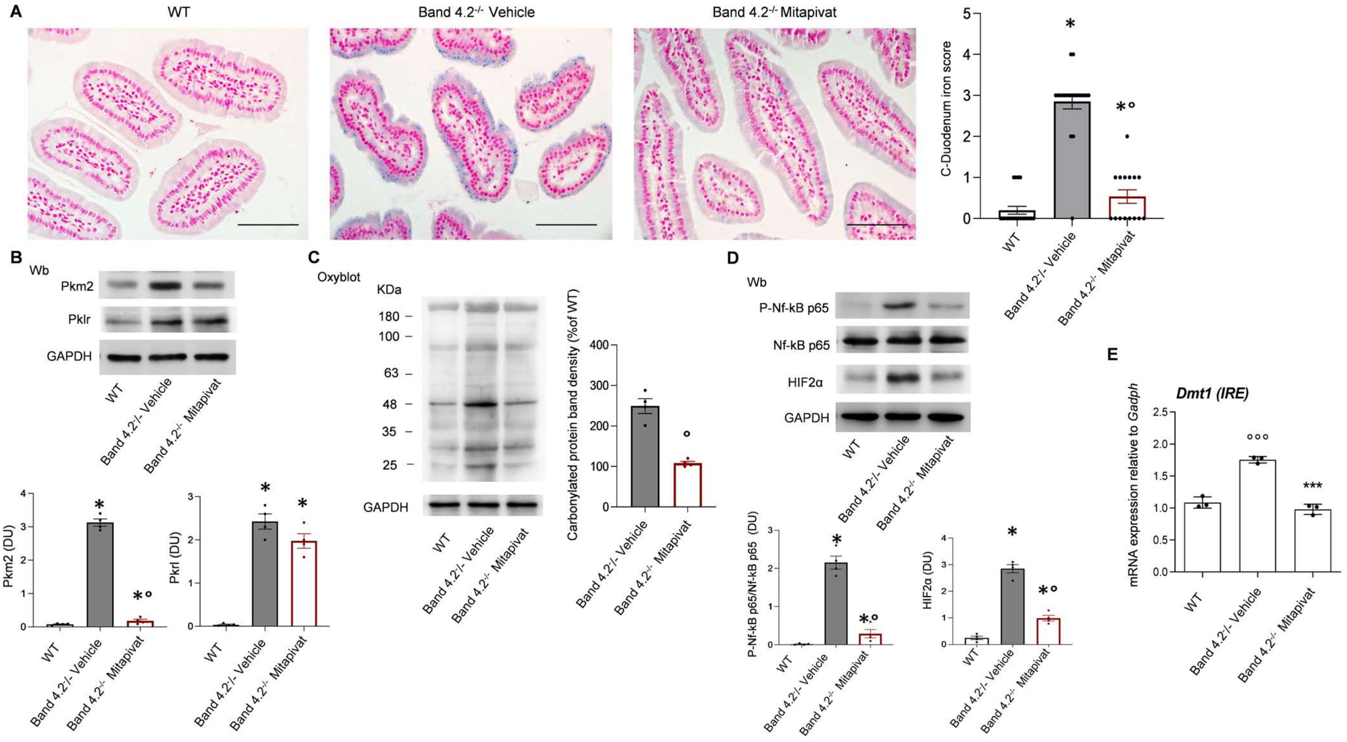

Figure 9S. A. Iron staining (Perl's Prussian blue is a semi-quantitative method to assess organ iron accumulation) in duodenum from WT and 4.2$^{-/-}$ mice treated with either vehicle or mitapivat (100 mg/Kg/day) for 6 months. One representative image for each group is shown. Data are mean ± SEM. * P < 0.05 compared with WT mice and ° P < 0.05 compared with vehicle-treated mice by t-test. **B.** Western-analysis (Wb) using specific antibodies against PKM2 and PKLR in duodenum from WT and 4.2$^{-/-}$ mice treated as in A. 50 μgr of protein loaded on an 8% T, 2.5%C polyacrylamide gel. Gapdh serves as protein loading control. One representative gel from 4 with similar results is shown. **Lower panel.** Densitometric analysis of Western-blot (Wb). Data are presented as mean ±SEM (n=4), * P < 0.05 compared with WT mice and ° P < 0.05 compared with vehicle-treated mice by t-test. **C.** OxyBlot analysis of the soluble fractions of duodenum from WT and 4.2$^{-/-}$ mice treated as in A. The carbonylated proteins (1 mg) were detected by treating with 2,4-dinitrophenylhydrazine and blotted with anti-DNP antibody. Gapdh serves as protein loading control. Quantification of band area is shown in the **right panel.** Data are presented as mean ±SEM (n=3), ° P < 0.05 compared with vehicle-treated mice by t-test. **D.** Western-blot (Wb)analysis using specific antibodies against phosphorylated (p-)NF-κB p65, NF-κB p65 and HIF 2 α in duodenum from WT and 4.2$^{-/-}$ mice treated as in A. 75 μgr of protein loaded on an 8% T, 2.5%C polyacrylamide gel. Gapdh serves as protein loading control. One representative gel from 4 with similar results is shown. Densitometric analysis of immunoblots is shown in the lower panel. Data are presented as mean ±SEM (n=4), * P < 0.05 compared with WT mice and ° P < 0.05 compared with vehicle-treated mice by t-test. **E.** mRNA expression of Dmt1 (IRE) by qRT-PCR of duodenum from WT and 4.2$^{-/-}$ mice treated as in A. Experiments were performed in triplicate. Data are mean ± SD (n=3). °°°P < 0.01, 4.2$^{-/-}$ vehicle vs WT mice; ***P < 0.01 4.2$^{-/-}$ vehicle vs mitapivat treated mice. P value was calculated by ANOVA, internal comparisons by post-hoc correction by Tukey's multiple comparisons test.

Figure 10S

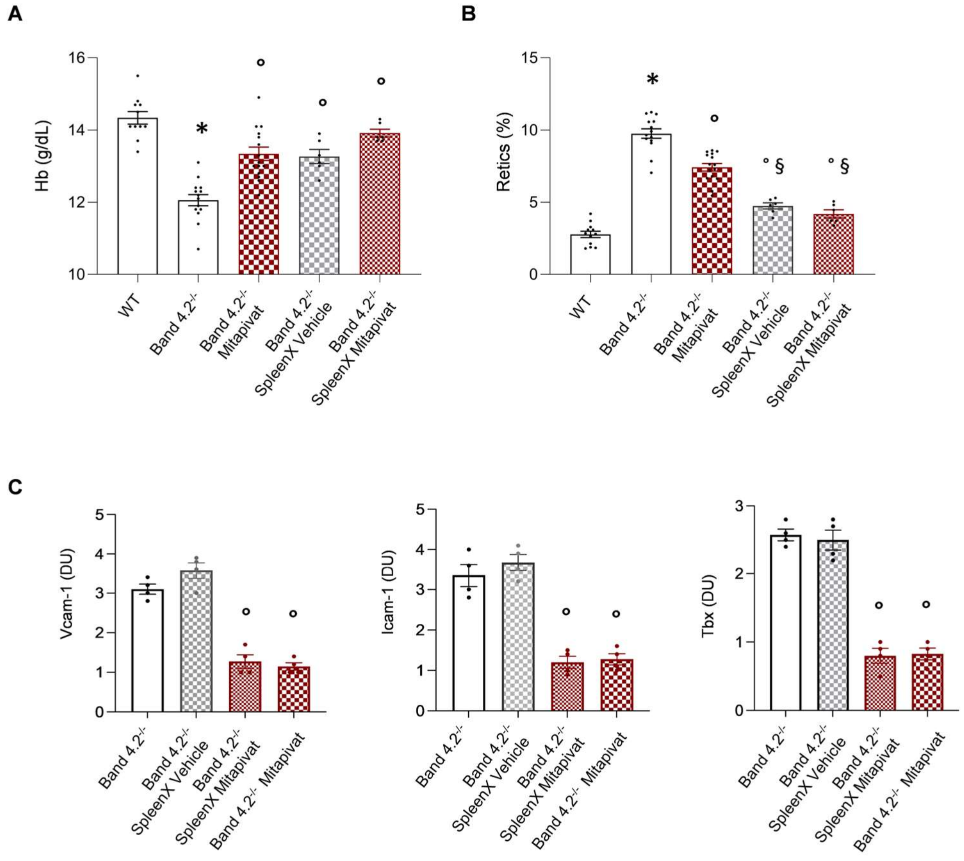

Figure 10S. A-B. Hemoglobin and reticulocyte count of WT and 4.2$^{-/-}$ mice splenectomized (SpleenX) or not treated with vehicle or mitapivat (100 mg/Kg/day) up to 7 months of age. Data are means ± SEM (n=6-14). * $P < 0.05$ compared with WT mice, ° P< 0.05 compared to vehicle treated 4.2$^{-/-}$ mice; § $P < 0.05$ compared to mitapivat treated 4.2$^{-/-}$ mice by two-way ANOVA. **C.** Densitometric analysis of Western-blots as in Figure 6F. Data are presented as mean ±SEM (n=4), ° $P < 0.05$ compared with vehicle-treated mice by t-test.
